# Reprofiling of pyrimidine-based DAPK1/CSF1R dual inhibitors: identification of 2,5-diamino-4-pyrimidinol derivatives as novel potential anticancer lead compounds

**DOI:** 10.1080/14756366.2019.1699554

**Published:** 2019-12-06

**Authors:** Ahmed K. Farag, Ahmed H. E. Hassan, Byung Sun Ahn, Ki Duk Park, Eun Joo Roh

**Affiliations:** a RI Translational Research Team, Division of Applied RI, Korea Institute of Radiological and Medical Sciences (KIRAMS), Seoul, Republic of Korea; b Department of Medicinal Chemistry, Faculty of Pharmacy, Mansoura University, Mansoura, Egypt; c Chemical Kinomics Research Center, Korea Institute of Science and Technology (KIST), Seoul, Republic of Korea; d Division of Bio-Medical Science and Technology, KIST School, University of Science and Technology, Seoul, Republic of Korea; e Convergence Research Center for Diagnosis, Treatment and Care System of Dementia, Korea Institute of Science and Technology (KIST), Seoul, Republic of Korea

**Keywords:** Reprofiling, anticancer, kinase inhibitors, DAPK1, CSF1R, PAMPA assay

## Abstract

Hybridization of reported weakly active antiproliferative hit 5-amino-4-pyrimidinol derivative with 2-anilino-4-phenoxypyrimidines suggests a series of 2,5-diamino-4-pyrimidinol derivatives as potential antiproliferative agents. Few compounds belonging to the proposed series were reported as CSF1R/DAPK1 inhibitors as anti-tauopathies. However, the correlation between CSF1R/DAPK1 signalling pathways and cancer progression provides motives to reprofile them against cancer therapy. The compounds were synthesised, characterized, and evaluated against M-NFS-60 cells and a kinase panel which bolstered predictions of their antiproliferative activity and suggested the involvement of diverse molecular targets. Compound **6e**, the most potent in the series, showed prominent broad-spectrum antiproliferative activity inhibiting the growth of hematological, NSCLC, colon, CNS, melanoma, ovarian, renal, prostate and breast cancers by 84.1, 52.79, 72.15, 66.34, 66.48, 51.55, 55.95, 61.85, and 60.87%, respectively. Additionally, it elicited an IC_50_ value of 1.97 µM against M-NFS-60 cells and good GIT absorption with P_e_ value of 19.0 ± 1.1 × 10^−6 ^cm/s (PAMPA-GIT). Molecular docking study for **6e** with CSF1R and DAPK1 was done to help to understand the binding mode with both kinases. Collectively, compound **6e** could be a potential lead compound for further development of anticancer therapies.

## 1. Introduction

Cancer persists to be a major hurdle to human wellbeing causing the second-highest mortality rate after cardiovascular diseases^1^. Despite the advances in chemotherapeutic approaches, unmet clinical needs maintain cancer as a leading cause of death^2^^,^^3^. Furthermore, cancer is a group of heterogeneous diseases with multifactorial etiology and inevitability for resistance evolvement. These challenges along with our continuous understanding of oncogenesis suggest that a multitarget single molecule with polypharmacological mechanisms might afford a novel promising chemotherapeutic agent^4^.

**Figure 1.**
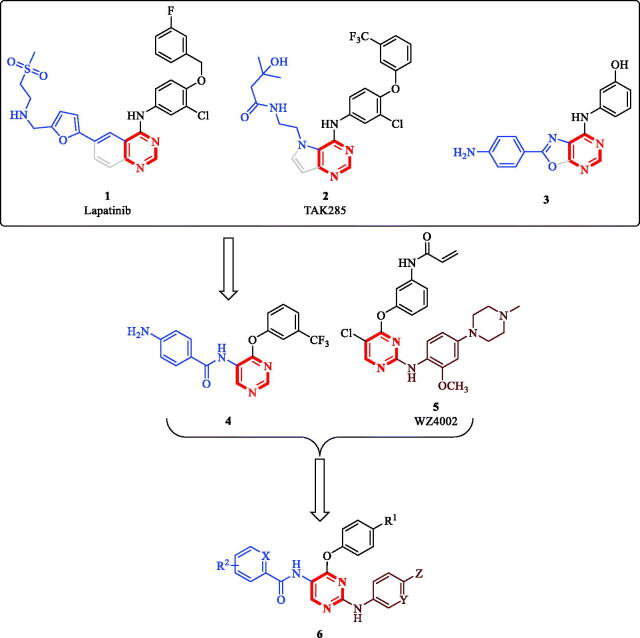
Reported pyrimidine-based anticancer molecules and design rationale for anticancer 2,5-diamino-4-pyrimidinol derivatives.

Substituted and/or fused bicyclic pyrimidine-containing scaffolds are privileged structures in medicinal chemistry with diverse bioactivities and success stories reported including the development of anticancer, anti-inflammatory, antiviral and antibacterial pyrimidine-based chemical entities^5–13^. Starting from fused-pyrimidine scaffolds known in anticancer agents, such as lapatinib (**1**, Figure 1), TAK285 (**2**, Figure 1), compound **3** (Figure 1), our team has identified 5-amino-4-pyrimidinol hit compound **4** out of a small library. This library was originally developed by decomposing the ring fused to the pyrimidine ring while maintaining the relative positions of the other substituents (Figure 1)^14^. While screening the reported generated library for anticancer activity, we have noticed that the derivatives having a chloro substituent at the 2-position of the pyrimidine core possessed slightly enhanced antiproliferative activity; yet they lacked EGFR inhibitory activity. Additionally, among unfused anticancer pyrimidines, literature reports promoted 2,4-diarylpyrimidine derivatives, such as WZ4002 (**5**, Figure 1), to have excellent antiproliferative activity^15^. Consequently, we thought of using hit compound **4** as a starting point to design hybrid compounds combining the pharmacophoric features of both hit compound **4** and the 2-amino-4-pyrimidinol-based 2,4-diarylpyrimidine derivatives. Thus, in addition to the 4-phenoxy feature shared by both hit compound **4** and WZ4002, the offspring molecules (**6**, Figure 1) would also inherit the 5-benzamide feature from hit compound **4** and the 2-anilino feature from WZ4002. Noteworthy, some recently reported non-ATP-competitive DAPK1, ATP-competitive CSF1R kinases dual inhibitors for the treatment of the neurodegenerative tauopathies possess similar structures to this design rationale even though they were designed via different approach and for a different therapeutic purpose^16^. Therefore, reprofiling the bioactivity of such compounds against cancer cells was strongly encouraged. Reprofiling i.e. repurposing or repositioning is a powerful tool of modern medicinal chemistry that offers many advantages and resulted in several successful outcomes^17–20^.

Death-Associated Protein Kinase-1 (DAPK1), which controls several functions of the human cell, is well-known for its role in apoptosis, autophagy, and suppressing tumour growth^21^. However, depending on the cellular context and cellular stimulants, it might operate to promote or suppress cellular growth^21–23^. Interestingly, DAPK1 Knockdown or inhibition significantly suppressed the growth of the HCC1143, HCC1937, HCC1954 triple-negative breast cancer (TNBC) cell lines by 80–90% suggesting the development of DAPK1 inhibitors to target TNBC^24^. In another report, silencing DAPK1 by siRNA in HHUA human endometrial adenocarcinoma cell line increased the apoptotic cell death. These results suggest the use of DAPK1 inhibitors for targeting uterine cancers^25^^,^^26^. Besides, ZIP kinase (DAPK3) is activated by DAPK1, which makes DAPK1 inhibitors potential downregulators of ZIP kinase activity^27^. As the activity of the downstream ZIP kinase is critically required in several cancers including prostate, colon and lung cancers, inhibitors of the upstream DAPK1 activity could be beneficial for the treatment of multiple types of cancer^28–30^.

On the other hand, CSF1R kinase and/or its ligands are overexpressed in several human cancers including anaplastic large cell lymphoma (ALCL), classical Hodgkin’s lymphoma (cHL), breast, ovarian, and prostate cancers in addition to controlling the proliferation and survival of tumour-associated macrophages (TAMs)^31–35^. Blocking CSF1R signalling with recombinant antibody-induced apoptosis of cancer cells, depleted TAMs and delayed tumour growth and metastasis^36–38^. Recently, it was proven that the overexpression of the natural ligands of CSF1R (CSF1 or IL-34) is associated with tumour progression and poor survival in lung cancer patients^39^. In addition, TAMs overexpressing CSF1R correlate with poor prognosis and poor overall survival in several human cancers^40–43^. Collectively, these reports and the above-mentioned design provide a solid rationale and offer strong motives to synthesise molecules possessing the chemical structure **6** and reprofile them against cancer cells. The compounds being investigated in this report are illustrated in Table 1 and the results of this study are reported in the following sections.

**Table 1.** General structure and specific examples of the compounds being evaluated for their antiproliferative effects in this report.


Compound	R^1^	R^2^	X	Y	Z
**6a**	OMe	3,5-Dimethoxy	CH	CH	Morpholino
**6b**	OMe	3-MeO	CH	CH	Morpholino
**6c**	OMe	3-CF_3_	CH	CH	Morpholino
**6d**	OMe	3,5-Dimethoxy	CH	N	Morpholino
**6e**	OMe	3,5-Dimethoxy	CH	N	Piperidino
**6f**	OMe	3,5-Dimethoxy	CH	CH	Fluoro
**6g**	CF_3_	3,5-Dimethoxy	CH	N	Morpholino
**6h**	CF_3_	H	N	N	Morpholino

## 2. Materials and methods

### 2.1. Chemistry

All manipulations and reactions were performed using standard Schlenk techniques. The starting materials, reagents, and solvents were obtained from commercial suppliers and were used without further purification. Thin-layer chromatography was performed with Merk silica gel 60 F_254_ pre-coated glass sheets. Column chromatography was performed on Biotage Isolera One™ flash chromatography system and the eluting solvents are noted as a mixed solvent with given volume-to-volume ratios or as a percentage. Uncorrected melting points were measured using Optimelt Automated Melting Point System (Stanford Research Systems). ^1^H and ^13^C NMR were measured on a 400 MHz Bruker Avance or a 500 MHz Agilent 500 NMR spectrometer. Chemical shifts and coupling constants are presented in parts per million (ppm) relative to Me_4_Si and hertz (Hz), respectively, and the following abbreviations are used: s, singlet; d, doublet; dd, a doublet of doublets; t, triplet; m, multiplet. High-resolution mass spectra were performed on Waters ACQUITY UPLC BEH C18 1.7µ−Q-TOF SYNAPT G2-Si High Definition Mass Spectrometry.

### 2.2. General procedure for preparation of 8, 9, and 6g–h

The procedure for the preparation of **6g–h** was adapted following the reported protocol^8^.

#### General procedure for preparation of 8, and 9

##### *N*-(6-morpholinopyridin-3-yl)-5-nitro-4–(4-(trifluoromethyl)phenoxy)pyrimidin-2-amine (8)

Compound **8** was prepared by adding a THF solution (3 ml) of 6-morpholinopyridin-3-amine (1 eq) to a THF solution (5 ml) of compound **7** followed by pyridine (1 eq) at 0 °C. The mixture was allowed to warm to room temperature and stirred overnight. After reaction completion, the mixture was evaporated to dryness and the residue was purified by column chromatography using 10–40% ethyl acetate in hexane to obtain compound **8** as yellow solid, yield 41.0%, MP: 196.8–197.3 °C. ^1^H NMR (400 MHz, CDCl_3_): δ 9.15 (s, 1H), 7.89 (d, 1H, *J* = 2.2 Hz), 7.75 (d, 2H, *J* = 8.4 Hz), 7.31 (d, 2H, *J* = 8.3 Hz), 7.17 (dd, 1H, *J* = 9.2, 2.4 Hz), 6.19 (d, 1H, *J* = 9.1 Hz), 3.80 (t, 4H, *J* = 4.4 Hz), 3.40 (t, 4H, *J* = 4.8 Hz); ^13^C NMR (100 MHz, CDCl_3_): δ 163.01, 159.63, 159.13, 158.80, 156.78, 154.55, 140.08, 130.20, 128.86, 128.53, 127.11, 127.08, 124.71, 124.15, 123.06, 122.64, 106.60, 105.68, 66.64, 45.52. HRMS (ES^+^): *m/z* calculated for C_20_H_17_F_3_N_6_O_4_: 463.1336 [M + H]^+^. Found 463.1333.

##### *N*^2^-(6-morpholinopyridin-3-yl)-4–(4-(trifluoromethyl)phenoxy)pyrimidine-2,5-diamine (9)

Compound **9** was prepared by dissolving compound **8** (1 mmol) in methanol (50 ml) followed by addition of 10% Pd/C (0.1 mmol) and the reaction was stirred overnight under a hydrogen atmosphere. The mixture was filtered through Celite and the filtrate was evaporated to dryness to obtain **9** that was used without further purification. Brownish white solid, yield 99.0%, MP: >250 °C. ^1^H NMR (500 MHz, DMSO-d_6_): δ 8.66 (s, 1H), 8.13 (dd, 1H, *J* = 2.8, 0.7 Hz), 7.86 (s, 1H), 7.83–7.76 (m, 2H), 7.56 (dd, 1H, *J* = 9.1, 2.8 Hz), 7.45–7.39 (m, 2H), 6.49 (d, 1H, *J* = 9.1 Hz), 4.62 (s, 2H), 3.64 (t, 4H, *J* = 5.7 Hz), 3.21–3.19 (m, 4H); ^13^C NMR (125 MHz, DMSO-d_6_): δ 158.11, 156.84, 154.66, 152.01, 144.33, 138.26, 130.33, 128.81, 127.50, 123.38, 123.24, 106.91, 66.48, 46.47, 40.64, 40.55, 40.47, 40.38, 40.30, 40.21, 40.13, 40.04, 39.97, 39.88, 39.71, 39.54. HRMS (ES^+^): *m/z* calculated for C_20_H_19_F_3_N_6_O_2_: 433.1594 [M + H]^+^. Found 433.1595.

#### General procedure for preparation of compounds 6g–h

Compound **9** (0.1 mmol) was dissolved in anhydrous DCM (5 ml) and cooled to −78 °C. To this solution was added a solution of the appropriate acid chloride (1.1 eq) in anhydrous DCM (2 ml) dropwise at −78 °C and the mixture was allowed to stir for 30 min at this temperature. The mixture was then allowed to warm up to room temperature and stirred overnight. After complete consumption of the amine as indicated by TLC, the solvent was evaporated, and the residue was purified with flash column chromatography using 20–50% ethyl acetate in hexane as the mobile phase to obtain **6g–h** as solids.

##### 3,5-dimethoxy-*N*-(2-((6-morpholinopyridin-3-yl)amino)-4–(4-(trifluoromethyl)phenoxy)pyrimidin-5-yl)benzamide (6 g)

White solid, yield 49.0%, MP: 199.6–201.6 °C. ^1^H NMR (400 MHz, CDCl_3_): δ 9.21 (s, 1H), 8.01 (d, 1H, *J* = 2.7 Hz), 7.88 (s, 1H), 7.73–7.70 (m, 2H), 7.52–7.49 (m, 1H), 7.29 (d, 2H, *J* = 8.4 Hz), 7.01 (d, 2H, *J* = 2.3 Hz), 6.84 (s, 1H), 6.63 (t, 1H, *J* = 2.2 Hz), 6.38 (d, 1H, *J* = 9.1 Hz), 3.85 (s, 6H), 3.80 (t, 4H, *J* = 4.9 Hz), 3.48 (t, 4H, *J* = 5.1 Hz); ^13^C NMR (100 MHz, CDCl_3_): δ 170.47, 165.35, 161.12, 160.46, 160.13, 157.90, 156.25, 156.06, 155.79, 154.73, 152.09, 151.64, 146.40, 142.57, 139.55, 138.18, 136.64, 136.1, 130.23, 129.32, 128.35, 128.02, 127.30, 126.97, 126.93, 126.79, 126.67, 126.63, 125.29, 122.88, 122.59, 121.27, 112.59, 106.73, 106.48, 106.39, 105.20, 103.89, 103.46, 66.74, 66.70, 55.67, 55.44, 46.0, 45.58. HRMS (ES^+^): *m/z* calculated for C_29_H_27_F_3_N_6_O_5_: 597.2068 [M + H]^+^. Found 597.2066.

##### *N*-(2-((6-morpholinopyridin-3-yl)amino)-4–(4-(trifluoromethyl)phenoxy)pyrimidin-5-yl)picolinamide (6h)

Brown solid, yield 41.0%, MP: 225.2–227.1 °C. ^1^H NMR (400 MHz, CDCl_3_): δ 10.13 (s, 1H), 9.38 (s, 1H), 8.65–8.63 (m, 1H), 8.30 (d, 1H, *J* = 7.8 Hz), 8.03 (d, 1H, *J* = 2.6 Hz), 7.95–7.91 (m, 1H), 7.74 (d, 2H, *J* = 8.5 Hz), 7.54 (d, 1H, *J* = 7.7 Hz), 7.52–7.49 (m, 1H), 7.36 (d, 2H, *J* = 8.3 Hz), 7.03 (s, 1H), 6.39 (d, 1H, *J* = 9.2 Hz), 3.82 (t, 4H, *J* = 4.8 Hz), 3.39 (t, 4H, *J* = 5.0 Hz); ^13^C NMR (100 MHz, CDCl_3_): δ 162.18, 159.97, 156.02, 155.54, 154.96, 151.01, 149.26, 148.29, 139.47, 137.73, 130.17, 128.25, 127.93, 127.44, 126.92, 126.89, 126.68, 125.33, 122.98, 122.63, 122.45, 112.87, 106.41, 66.75, 46.10. HRMS (ES^+^): *m/z* calculated for C_26_H_22_F_3_N_7_O_3_: 538.1809 [M + H]^+^. Found 538.1811.

### 2.3. Biological evaluations

#### 2.3.1. *In vitro* kinase assay

The assay was performed using “HotSpot” assay platform from Reaction Biology Corp^44^^,^^45^.

#### 2.3.2. *In vitro* antiproliferative assay using M-NFS-60 cell lines

The experimental details are discussed in the Supporting material.

#### 2.3.3. *In vitro* antiproliferative assay using NCI-60 cell lines

The assay was performed using the standard National Cancer Institute (NCI) protocol^46^.

#### 2.3.4. PAMPA-GIT assay:

The experimental details are discussed in the supporting material.

## 3. Results and discussion

### 3.1. Chemistry

**Scheme 1.**
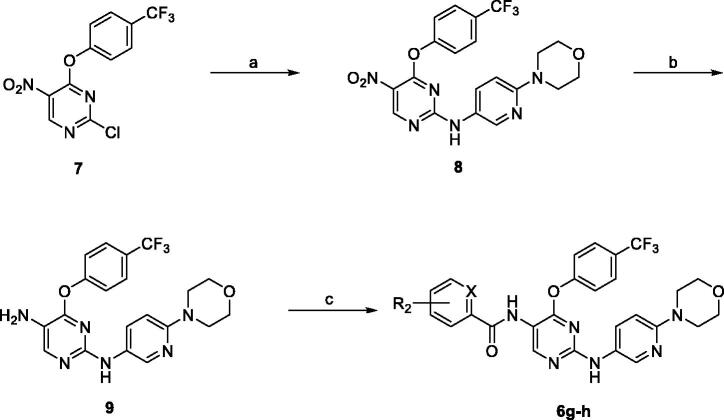
Synthesis of compounds **6g–h**. Reagents and conditions: (a) 6-morpholinopyridin-3-amine, pyridine, THF, rt, 12 h; (b) H_2_, 10% Pd/C, MeOH, rt, 12 h; (c) appropriate acyl chloride, DIPEA, DCM, −78 °C to rt, 2 h.

The reported compounds (**6a–f**) were resynthesised following the reported procedure^16^. The new compounds **6g** and **6h** were obtained by reacting 2-chloro-5-nitro-4–(4-(trifluoromethyl)phenoxy)pyrimidine (**7**) with 2-morpholino-5-aminopyridine in THF at room temperature to obtain compound **8** which was reduced by catalytic hydrogenation to obtain the amino derivative **9**, which was reacted with 3,5-dimethoxybenzoyl chloride in DCM and DIPEA to obtain **6g** or with α-picolinlyl chloride in DCM and pyridine to obtain **6h**. The structures of the new compounds were fully elucidated by ^1^HNMR, ^13^CNMR, and HRMS, and the experimental details are summarised in the experimental section (Scheme 1).

### 3.2. Biological evaluations

#### 3.2.1. Initial assessment against M-NFS-60 cell line

To explore whether this series triggers antiproliferative activity, selected compounds of the general skeleton **6** were initially assessed using M-NFS-60 mouse myelogenous leukaemia cells, which is a virus-induced lymphoblastoid murine cancer cell that overexpresses CSF1R, prior to reprofiling against human cancer cells. As shown in Table 2, the 10 µM dose of compounds **6a–e** triggered significantly high growth inhibition of the M-NFS-60 cells. Compounds **6e** and **6b** showed the highest measured growth inhibition values by 99.2 and 92.3% while compound **6c** was less effective showing 52.6% growth inhibition. Interestingly, attempts to relate the previously known kinase inhibition data of compounds **6a–h** revealed that the best growth inhibitor, compound **6e**, possessed less CSF1R and DAPK1 inhibitory activities relative to the second most active M-NFS-60 growth inhibitor compound **6b** (Table 2). In fact, **6d** is 2.5-folds less potent than **6e** as an M-NFS-60 growth inhibitor, even though compounds **6e** and **6d** had a similar CSF1R/DAPK1 inhibition profile. In addition, compound **6b**, despite the high activity as a DAPK1 inhibitor, possessed a similar CSF1R inhibitory activity to compound **6c** that was the least active as M-NFS-60 growth inhibitor (Table 2). These results might suggest a partial contribution of CSF1R and DAPK1 inhibition to the overall elicited activity while other unknown targets might be involved in mediating the antiproliferative activities of these compounds. The results of this initial antiproliferative assay, though conducted employing non-human cancer cell, raised hopes that this class of compounds might possess potential anticancer activities. Therefore, we were encouraged to investigate the underlying molecular targets and to profile the series against human cancers.

**Table 2.** GI_50_ values (μM) exhibited by selected compounds over M-NFS-60 murine myeloblastic leukaemia cell line.

Compound	M-NFS-60 cell line	CSF1R	DAPK1
	% Inhibition^a^ (GI_50_ in μM^b^)	Inhibition (%)^a^	Inhibition (%)^a^
**6a**	77.87 ± 0.46 (5.81 ± 1.03)	95.34 ± 1.28	65.95 ± 2.21^c^
**6b**	92.31 ± 0.8 (4.10 ± 1.08)	91.85 ± 1.31	75.06 ± 1.26
**6c**	52.57 ± 0.61^c^ (10.2 ± 1.15)	74.70 ± 1.28	69.70 ± 1.19^c^
**6d**	88.81 ± 1.38 (4.96 ± 1.11)	77.94 ± 0.52	66.22 ± 0.26
**6e**	99.18 (1.97 ± 1.40)	75.99 ± 0.49	67.28 ± 2.95
**6f**	ND	35.20	65.00
**6g**	ND	ND	17.16 ± 2.10
**6h**	ND	ND	7.12 ± 2.98

^a^ % Inhibition ± SD value at 10 μM dose.

^b^ GI_50_±SD value data were generated from the dose-response curve plotted from a 10-dose experiment starting at 10 μM performed in duplicate.

^c^ Measured at 20 μM.

ND: Not determined.

#### 3.2.2. Kinase panel assay

The initial assessment of cytotoxicity on the M-NFS-60 cell line uncovered that this class of compounds has significant antiproliferative activity. Among them, compound **6e** was the most potent, therefore, we thought of evaluating its inhibition activity and selectivity against a panel of kinases.

The kinase panel consisted of 14 different kinases correlated with cancer and representing diverse kinase families and groups. The employed kinase panel included EGFR as the starting hit compound was found as an EGFR inhibitor. In contrast to hit compound **4**, compound **6e** was unable to inhibit EGFR as shown by the results in Table 3. In addition, compound **6e** did not inhibit the kinase reaction of any of AXL, c-MER, CDK2/cyclin A, FLT1/VEGFR1, JAK3, KDR/VEGFR2, PDGFRa, PDGFRb, RET, TYRO3/SKY, and c-MET kinases. Only c-Kit and FLT3 kinases were poorly inhibited by compound **6e**. Accordingly, by considering the moderate enzyme inhibition ability of **6e** against DAPK1 and CSF1R and the relatively higher potency in the cellular assay, we anticipate that DAPK1 and CSF1R inhibitions could be partial drivers to the observed cellular activity and that there could be other molecular target(s) involved.

**Table 3.** % Inhibition of kinase reactions of a panel of kinases by 10 μM doses of compound **6e**.

Kinase	% Inhibition	Kinase	% Inhibition
AXL	0.44	**FLT3**	**14.52**
**c-Kit**	**3.63**	JAK3	−16.66
c-MER	−5.95	KDR/VEGFR2	−16.61
c-MET	−16.40	PDGFRa	−5.94
CDK2/cyclin A	−7.09	PDGFRb	−1.76
EGFR	−9.78	RET	−3.48
FLT1/VEGFR1	−5.79	TYRO3/SKY	−10.84

The significance of bold values represent the highest values.

#### 3.2.3. Profiling against various human cancer diseases

Profiling was conducted against human cancer cells after the initial assessment of the antiproliferative activity of the selected compounds against M-NFS-60 cells suggested that this type of compounds could be possible candidates to develop novel antiproliferative chemical entities. Because cancer is not a single disease but a heterogeneous group of diseases that might originate from diverse body tissues, bioactivity screening was conducted using nine panels of cancer diseases employing the well-trusted sulforhodamine B (SRB) assay protocol.

##### 3.2.3.1. Profiling against human hematological cancers

**Figure 2.**
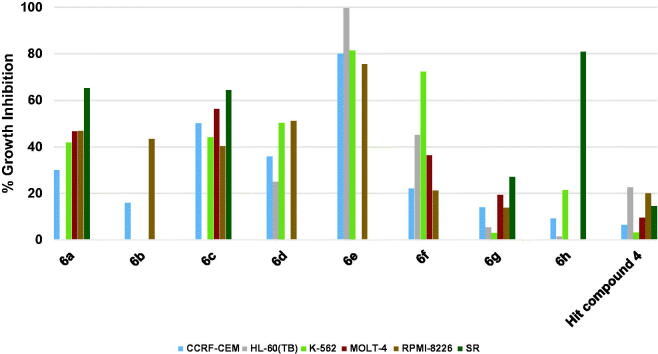
Measured % growth inhibition of various hematological cancers triggered by 10 µM doses of compounds **6a–h** and hit compound **4**. Values are the averages of duplicate assays.

The results of profiling against six different hematological cancers showed that the highest growth inhibition triggered by the starting point hit compound **4** was around 20% against two cell lines while the triggered growth inhibition was weaker against the remaining four cell lines (Figure 2). When the trifluoromethyl substituent on the phenoxy moiety of hit compound **4** was shifted to the 4-position, a picolinamido moiety replaced the 4-aminobenzamido moiety and 6-morpholinopyridin-3-ylamino was introduced at 2-position of the pyrimidine core, compound **6h** was obtained that showed potent inhibitory activity against SR cell line (80.74% growth inhibition, Figure 2). Surprisingly, compound **6h** showed no or weak inhibition against other hematological cancer cell lines indicating an excellent selectivity to the SR cell line. Replacing the picolinamido moiety of compound **6h** with 3,5-dimethoxybenzamido afforded compound **6g**, which showed poor activity similar to the starting hit compound **4**. Furthermore, compound **6f** in which the phenoxy moiety bears a 4-methoxy substituent, 3,5-dimethoxybenzamide moiety maintained and 4-fluoroanilino moiety is affixed at 2-position of the pyrimidine ring showed enhanced activity relative to hit compound **4** against all cell lines, especially against K-562 cell line (72.24% growth inhibition, Figure 2). Replacement of the 4-fluoroanilino moiety of compound **6f** with 4-morpholinoanilino resulted in further enhancement of the inhibitory activity of compound **6a** against almost all the tested hematological cancer cell lines (Figure 2). Removal of one the methoxy groups from the benzamide moiety of compound **6a** resulted in attrition of the activity of compound **6b** with only moderate activity against RPMI-8226 myeloma cell line. However, replacement of the 3-methoxy substituent on the benzamide moiety of compound **6b** with trifluoromethyl resulted in the significantly more active compound **6c** (Figure 2). Modifying the structure of compound **6a** through converting the phenyl ring of the anilino moiety into the heterocyclic pyridinyl ring afforded compound **6d** which did not show activity improvement relative to **6a**. Pleasantly, further modification of **6d** via converting the morpholino moiety into piperidino moiety afforded compound **6e** possessing excellent activities against all the tested hematological cancer cell lines. Interestingly, **6e** was able to completely inhibit the growth of the acute promyelocytic leukaemia HL-60(TB) cell line.

##### 3.2.3.2. Profiling against human lung cancers

**Figure 3.**
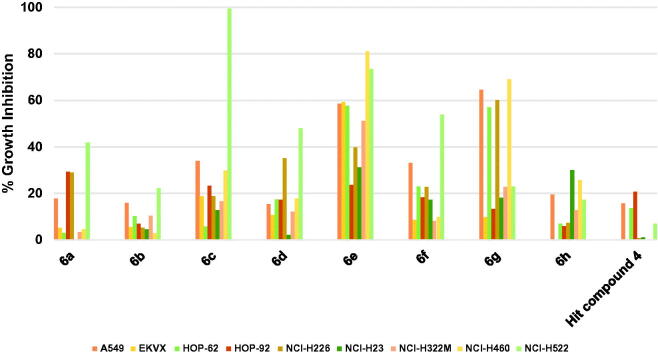
Measured % growth inhibition of various NSCLC cells triggered by 10 µM doses of compounds **6a–h** and hit compound **4**. Values are the averages of duplicate assays.

Profiling hit compound **4** against nine different non-small cell lung cancer (NSCLC) cell lines, as illustrated in Figure 3, showed a weak activity as only three cell lines were inhibited by 20.73–13.54% while the other cell lines were not significantly inhibited (Figure 3). Compound **6h** showed modest activity improvement relative to starting hit compound **4** (Figure 3). Compound **6g**, having 3,5-dimethoxybenzamide moiety instead of the picolinamide moiety of compound **6h**, showed significantly increased growth inhibition that approached or surpassed 60% (Figure 3) against four cell lines. However, the activity of **6g** against other lung cancer cell lines was around or below 20% growth inhibition. Modifying compound **6g** into compound **6f** resulted in significant lower activities against four cell lines (A549/ATCC, HOP-62, NCI-H226, and NCI-H460) relative to compound **6g**. Nevertheless, **6f** was more active than **6g** only in the case of NCI-H522 cell line (Figure 3). Replacement of the 4-fluoroanilino moiety of compound **6f** with 4-morpholinoanilino resulted in compound **6a** with lower activity against the tested NSCLC cell lines except for HOP-92 and NCI-H226 which were inhibited by slightly higher values relative to compound **6f**. Similar to the activity trend against hematological cancers, removal of one the methoxy groups from the benzamide moiety of compound **6a** resulted in attrition of activity of compound **6b**. Replacing the 3-methoxy substituent on the benzamide moiety of compound **6b** with 3-trifluoromethyl resulted in a significantly high increase of activity of compound **6c** against NCI-H522 NSCLC cell line (99.36% growth inhibition, Figure 3). However, the enhancement of activity against other cell lines was modest suggesting a high affinity for a vulnerable target specifically overexpressed in this cell line. Converting the phenyl ring of the anilino moiety of compound **6a** into a heterocyclic pyridinyl ring afforded compound **6d** which did not show significant activity improvement relative to **6a**. Further modification of **6d** via isosteric replacement of the oxygen atom of the morpholino moiety by methylene group afforded compound **6e**, which possessed significantly high growth inhibitory activities against seven of the tested NSCLC cell lines, which were near 80% growth inhibition against two cell lines and 60% against three cell lines (Figure 3).

##### 3.2.3.3. Profiling against human colorectal cancers

**Figure 4.**
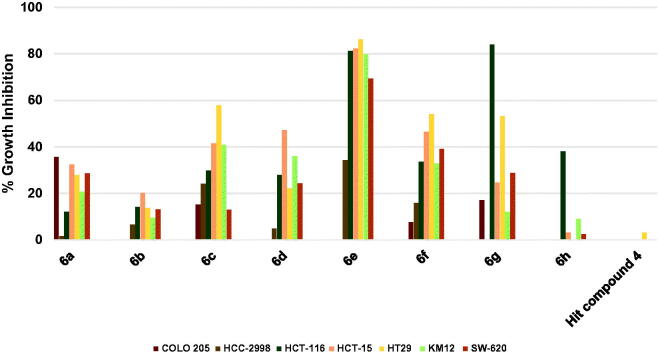
Measured % growth inhibition of various colon cancers triggered by 10 µM doses of compounds **6a–h** and hit compound **4**. Values are the averages of duplicate assays.

As shown in Figure 4, the hit compound **4** was ineffective against colon cancers, however compound **6h** showed significant, yet humble growth inhibition of HCT-116 cancer cells by 38.08%, albeit ineffective against other colon cancer cells (Figure 4). Interestingly, replacement of picolinamide moiety of compound **6h** by 3,5-dimethoxybenzamide moiety to afford compound **6g** resulted in activity enhancement against all cells, especially HCT-116 cell line which was inhibited by a high value of 83.91%, and HT29 cell line which was inhibited by 53.18%. Even though compound **6f** was derived from compound **6g** by replacing the 4-trifluoromethylphenoxy moiety by 4-methoxyphenoxy moiety and the pyridinylamino moiety by 4-fluoroanilino moiety, it was less active against HCT-116 cells. Despite that, **6f** maintained the same growth inhibition of **6g** against HT29 cells and elicited relatively higher growth inhibition values against most of the other tested colon cancer cells (Figure 4). Introducing the polar morpholino group to **6f** by replacing its fluoro atom resulted in compound **6a** which was less active compared to **6f** against six of the tested colon cancer cell lines. Further activity loss was found for compound **6b** which differs from **6a** by having 3-methoxy group instead of the 3,5-dimethoxy groups on the benzamide moiety. Unlike the activity trend observed in the majority of tested NSCLC cell lines, the increase in activity was more significant in the case of compound **6c** relative to **6b**. For instance, HT29 cells were significantly inhibited by 57.77% while the growth inhibition values of HCT-15 and KM12 cells were around 40% (Figure 4). In the case of **6d**, the positive change in activity was not high, despite that the growth inhibitory activity of HCT-15 cells approached 50% (Figure 4). Interestingly, a highly active compound **6e** was obtained by a minor structure modification of compound **6d**. To illustrate, **6e** triggered almost 80% growth inhibition against four colon cancer cell lines (HCT-116, HCT-15, HT29 and KM12; Figure 4) and around 70% growth inhibition against one colon cancer cell line (SW-620) while only HCC-2998 cell showed relatively low inhibition near 35% (it was not tested against COLO 205).

##### 3.2.3.4. Profiling against human brain cancers

**Figure 5.**
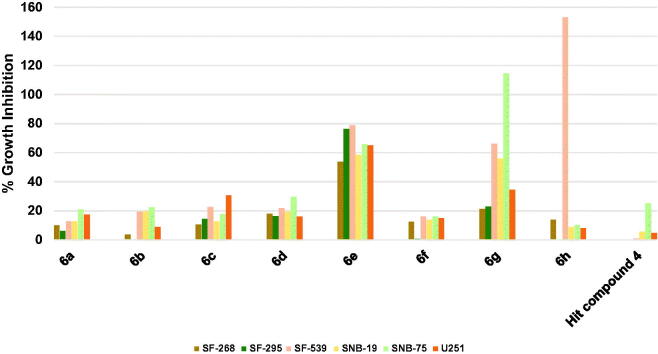
Measured % growth inhibition of various brain cancer cell lines triggered by 10 µM doses of compounds **6a–h** and hit compound **4**. Values are the averages of duplicate assays.

Except for SNB-75 brain cancer whose growth was inhibited by 25.17%, the hit compound **4** was ineffective against other brain cancers as depicted in Figure 5. Surprisingly, **6h**, which possesses 4-trifluoromethyl substituent on the phenoxy moiety, picolinamide moiety, and 6-morpholinopyridin-3-ylamino at 2-position of pyrimidine, showed a dramatic lethal activity against SF-539 with 153.05% growth inhibition value. Intriguingly, its activity against other brain cancers was comparatively negligible which gives the compound a high selectivity to the SF-539 cell line (Figure 5). Replacement of only picolinamide moiety of compound **6h** by 3,5-dimethoxybenzamide moiety resulted in encouraging activities of compound **6g** as it inhibited the growth of three brain cancer cell lines namely SNB-75, SF-539, and SNB-19 by 114.54, 66.02, and 55.94%, respectively. As illustrated in Figure 5, compounds **6a**–**d** and **6f** were significantly impotent as the growth inhibition was less than 20% against most of the tested six brain cancer cell lines. However, compound **6e** was comparatively highly active inhibiting all tested brain cancers by more than 50% despite the minor structural difference from compound **6d**, which might imply the presence of a possible activity cliff by this simple change. To exemplify, **6e** inhibited the growth of SF-295 and SF-539 cell lines by nearly 80%, SNB-75, and U251 cell lines by around 65%, SNB-19 by around 60% and finally SF-268 by around 55%. These results suggest compounds **6e**, **6g**, and **6h** as a leading compound for the development of potential CNS tumour therapies.

##### 3.2.3.5. Profiling against human melanoma cancers

**Figure 6.**
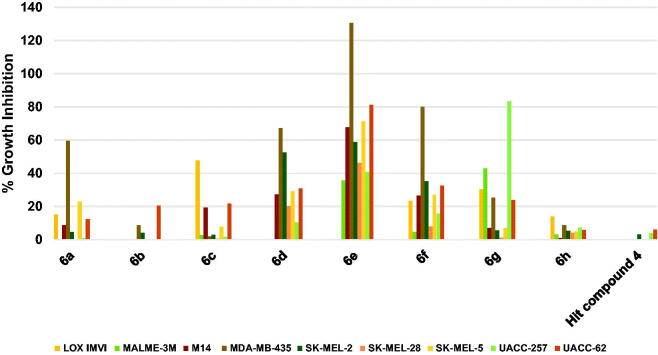
Measured % growth inhibition of various melanoma cell lines triggered by 10 µM doses of compounds **6a–h** and hit compound **4**. Values are the averages of duplicate assays.

Herein also, as illustrated in Figure 6, the hit compound **4** was of very low efficacy against the tested nine melanoma cell lines showing no inhibition against six cell lines and very weak activity that does not exceed 6% against the remaining three cell lines. Similarly, compound **6h** showed no significant inhibition of any of the nine melanoma cell lines (Figure 6). In addition, compound **6b** did not inhibit five cell lines but inhibited UACC-62 cells by around 20% (Figure 6). Other compounds triggered much more significant growth inhibition against melanoma cancers despite some compounds showed low growth inhibition against some melanoma cells. To illustrate, **6a** inhibited the growth of MDA-MB-435 cells by 59.6%; **6c** inhibited the growth of LOX IMVI by 47.6; **6d** inhibited the growth of MDA-MB-435 and SK-MEL-28 by 67.27 and 52.39%, respectively; **6f** inhibited the growth of MDA-MB-435 by a high value of 79.80%; and **6g** inhibited the growth of UACC-257 by 83.27% and MALME-3M by 42.79%. Similar to the above-mentioned results against other cancers, compound **6e** was the best hit among the tested compounds showing excellent activity against several cell lines (Figure 6). Compound **6e** lethally killed MDA-MB-435 cells by 130.59% growth inhibition, inhibited the growth of UACC-62 cells by 81.16%, SK-MEL-5 cells by 71.26%, M14 cells by 67.69%, and SK-MEL-2 by 58.78%. Accordingly, compound **6e** might serve as a leading compound for the development of potential anti-melanoma therapies.

##### 3.2.3.6. Profiling against human ovary cancers

**Figure 7.**
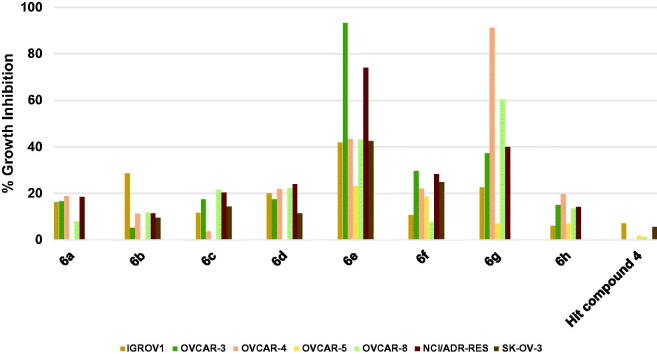
Measured % growth inhibition of various ovarian cancer cell lines triggered by 10 µM doses of compounds **6a–h** and hit compound **4**. Values are the averages of duplicate assays.

As illustrated in Figure 7, the hit compound **4** did not inhibit the growth of three ovarian cancer cell lines while the highest-recorded inhibition against other ovarian cancer cells was 7.04%. Profiling compounds **6a**–**d**, **6f**, and **6h** showed that these compounds elicited more significant growth inhibition relative to the starting hit compound albeit weak. To clarify, the maximum inhibition values for compound **6a** were around 18.7–16.2% against OVCAR-4, NCI/ADR-RES, OVCAR-3 and IGROV1 cells; for compound **6b** was 28.5% against IGROV1; for compound **6c** were 21.5–20.3 against OVCAR-8 and NCI/ADR-RES; for compound **6d** were 24.0–20.0% against NCI/ADR-RES, OVCAR-8, OVCAR-4 and IGROV1; for compound **6f** were 29.5–18.6% against OVCAR-3, NCI/ADR-RES, SK-OV-3, OVCAR-4 and OVCAR-5; and for compound **6h** were 19.7–13.4% against OVCAR-4, OVCAR-3, NCI/ADR-RES, and OVCAR-8. Interestingly, compounds **6e** and **6g** elicited excellent growth inhibition against several ovarian cancer cell lines. The structural differences between the two compounds are the presence of 4-methoxy substituent on the phenoxy moiety and the piperidino moiety in compound **6e** instead of the 4-trifluoromethyl and the morpholino moiety, respectively in compound **6g**. Thus, compound **6e** produced excellent inhibition of the growth of OVCAR-3 and NCI/ADR-RES cells by 93.3 and 73.9%, respectively, but the growth of OVCAR-4, OVCAR-8, and IGROV1 was less inhibited showing 43.3–41.7%. Interestingly, this inhibition profile was reversed in compound **6g** as it produced excellent inhibition of the growth of OVCAR-4 and OVCAR-8 by 91.2 and 60.3%, respectively, but the growth of OVCAR-3 and NCI/ADR-RES was less inhibited showing 39.9–37.2%.

##### 3.2.3.7. Profiling against human renal cancers

**Figure 8.**
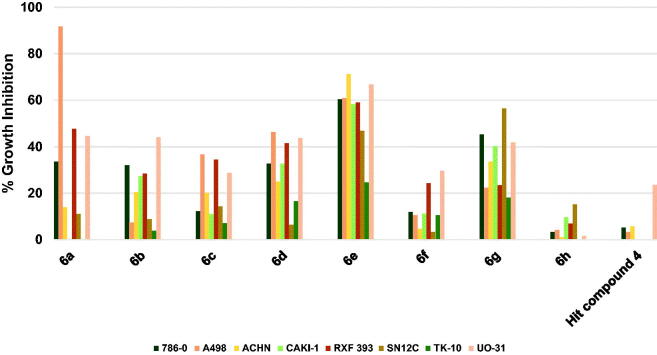
Measured % growth inhibition of various renal cancer cells triggered by 10 µM doses of compounds **6a–h** and hit compound **4**. Values are the averages of duplicate assays.

As Figure 8 illustrates, compound **4** inhibited the growth of UO-31 cells among the tested eight renal cancer cells slightly by 23.5% while the growth of other renal cancers was not inhibited or showed very low inhibition. The results of the profiling of compounds **6a**–**h** against different renal cancers showed that all compounds were more active relative to the starting hit compound **4**. While compound **6h** was of low activity showing less than 20% growth inhibition for all tested renal cancer cells, other compounds showed notable growth inhibition against several renal cancers. Among them, activity profiles of compounds **6a**, **6e** and **6g** were distinguished by pronounced activity. All three compounds share the same 3,5-dimethoxybenzamide moiety, while the 4-methoxyphenoxy moiety is common between compounds **6a** and **6e** and the morpholino moiety is common between compounds **6a** and **6g**. The activity profile of compounds **6a** elicited an exceptionally high inhibition of A498 cells by 91.7% in addition to average growth inhibition of RXF 393 and UO-31 cells by 47.7 and 44.5%, respectively. Meanwhile, compound **6g** triggered 56.4, 45.2, 41.9 and 40.3% growth inhibition of SN12C, 786-0, UO-31, and CAKI-1 cells, respectively. The best activity profile was detected for compound **6e** which produced good inhibition of six cell lines out of the tested eight renal cancers. To clarify, **6e** inhibited the growth of ACHN, UO-31, A498, 786-0, RXF 393 and CAKI-1 cells by 71.2–58.2%. From these results, it is inferred that compound **6e** might serve as a lead compound for the development of potential anti-renal cancers.

##### 3.2.3.8. Profiling against human prostate cancers

**Figure 9.**
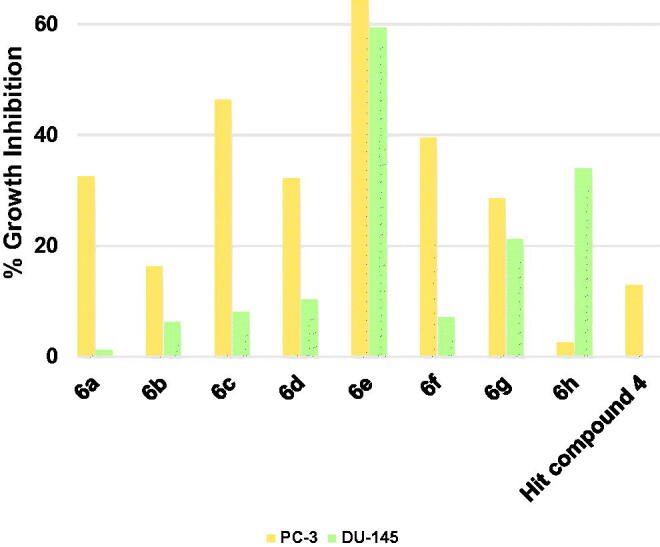
Measured % growth inhibition of various prostate cancer cell lines triggered by 10 µM doses of compounds **6a–h** and hit compound **4**. Values are the averages of duplicate assays.

The starting hit compound **4** triggered low inhibition of the prostate cancer PC-3 cells by 12.9% and did not show inhibition of DU-145 cells (Figure 9). Herein also compound **6e** was the best compound showing promising good inhibition of both PC-3 and DU-145 cells. Among other tested compounds, compound **6g** which showed a near balanced, albeit weak inhibition of both tested prostate cancers. There was a wide gap between the triggered growth inhibition of the two cell lines by all other compounds reflecting a difference in the tendencies of these compounds to inhibit both cells equally. Thus, compound **6a–d** and **6f** were more potential growth inhibitors of PC-3 cells rather than DU-145 cells (Figure 9). On the contrary, compound **6h** showed higher potentiality as an inhibitor of DU-145 cells. These results nominate compound **6e** to serve as a lead compound for the development of anti-prostate cancer therapies.

##### 3.2.3.9. Profiling against human breast cancers

**Figure 10.**
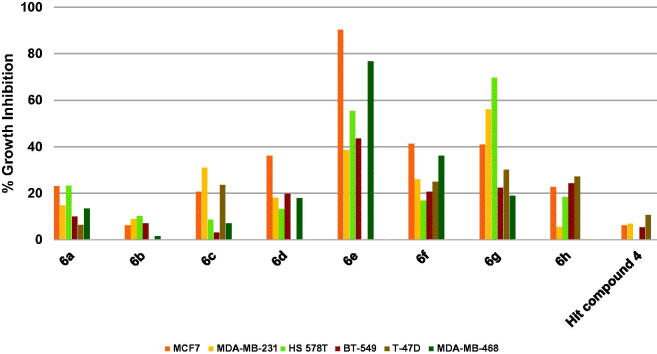
Measured % growth inhibition of various breast cancer cells triggered by 10 µM doses of compounds **6a–h** and hit compound **4**. Values are the averages of duplicate assays.

The results revealed that the starting hit compound **4** triggered low inhibition of breast cancers that did not exceed 10.7% (Figure 10). In contrast, compounds **6e** and **6g** were active eliciting excellent to good growth inhibition. For example, compound **6e** excellently inhibited the growth of the estrogen-dependent MCF7 cells by 90.2% as well as the TNBC MDA-MB-468 cells by 76.4%. It also triggered 55.5% growth inhibition of HS 578 T cells and 43.5% inhibition of BT-549 cells. Compound **6g**, which possesses 4-trifluoromethylphenoxy and morpholino moieties instead of the 4-methoxyphenoxy and piperidino moieties of the compound **6e**, was more active against HS 578 T cells eliciting 69.6% growth inhibition and also inhibited MDA-MB-231 cells by 56.0%. It is noteworthy that the excision of the cyclic amine piperidine moiety of compound **6e** to afford compound **6f**, which bears fluorine atom instead, resulted in diminished activity (Figure 10). The activities of other compounds (**6a**–**d** and **6h**) were lower relative to compounds **6e** and **6g**. These data suggest that among the tested compounds, compound **6e** might be advanced as a lead compound for further development of breast cancer therapies.

#### 3.2.4. Structure-activity relationship study

**Figure 11.**
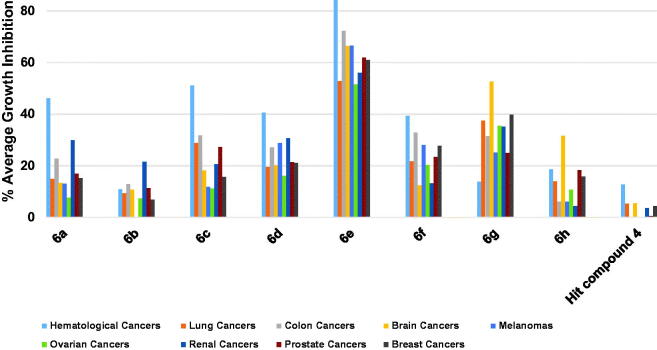
Average % values of measured growth inhibition of nine cancer diseases triggered by 10 µM doses of compounds **6a–h** and hit compound **4**. Values are the averages of duplicate assays.

Understanding the relationship between the observed activity and the underlying structural features of a certain series allows medicinal chemists to significantly improve the activity of this series. In our case, several conclusions can be drawn about the relation between the structural modifications and their effect on the antiproliferative activity as shall be discussed in this section. In contrast to the low activity of the hit compound **4**, the developed lead compound **6e** was able to inhibit the growth of multiple cancer cell lines, as illustrated in Figure 11. The structural features of compound **6e** involve 3,5-dimethoxybenzamide moiety, 4-methoxyphenoxy moiety and piperidino substituent on the pyridineamino moiety. These distinct structural features might be the reason behind the observed antiproliferative activity of **6e**. In this series, the piperidino moiety seems to be of crucial importance for a highly potent broad-spectrum antiproliferative agent. This could be inferred from the comparison of the activities of compounds **6e** and **6d** in which **6d** processes a morpholino group (Figure 1). One plausible explanation for this significant difference in the antiproliferative activity might be related to the hydrophobicity of the piperidine moiety^47^^,^^48^. This hydrophobicity might facilitate the desolvation of **6e** with respect to **6d**. Additionally, enhanced binding between **6e** and the host macromolecule might be gained from a potential entropy-driven water displacement at the binding site by the hydrophobic effect of the piperidine. In other words, the oxygen of the morpholino moiety of **6d** might encourage an unfavourable solvated state at the pocket of the target site, while the piperidine moiety displaces the water of solvation resulting in filling a hydrophobic pocket and gaining a higher binding affinity. Despite the notion that this simple isosteric replacement of the oxygen with methylene group (**6d** vs. **6e**) could results in drastic change in the whole molecular geometry and binding mode, the assumption that the two molecules bind the same pocket of a certain molecular target in the same binding fashion might make the solvation hypothesis plausible. In addition, the assumed higher affinity of **6e** for the host macromolecule(s) might reflect the higher antiproliferative activity at the cellular level.

The trade of carbon with a nitrogen atom at the 3-position of the 2-anilino moiety of the pyrimidine core was intended to allow for possible hydrogen bonding with the host molecule. This modification resulted in a slight improvement in the overall average antiproliferative activity (Figure 11), yet lead to a loss of the potency over the A498 renal cancer cell line. This is exemplified by comparing the activities of **6a** and **6d**. In fact, the dissymmetry of the pyridine ring compared with the phenyl ring could cause a change in the relative geometry of the morpholino group; it did not have a drastic effect on the activity as noticed with the **6d**-to-**6e** structural change.

In addition, compound **6f** is structurally different from compound **6e** by lacking the cyclic amine moieties, as well as the nitrogen atom of the heterocyclic pyridine ring which resulted in relatively lower activities (Figure 11). The influence of the 4-methoxy substituent on the phenoxy moiety might be deduced from the lower activity of compound **6g** possessing 4-trifluoromethyl substituent relative to compound **6d** (Figure 11). The positive influence of the 3,5-dimethoxy substitution pattern on the benzamide moiety might be concluded from comparing the activities of **6a** and **6b** in which **6b** possesses 3-methoxy substituent instead. Additionally, this influence can be understood by comparing compound **6g** with **6h** in which **6h** possesses 2-pyridineamide as a replacement to the benzamide moiety (Figure 11). However, comparing the activities of compound **6c**, possessing 3-trifluoromethyl with **6a** and **6b**, possessing 3,5-dimethoxy and 3-methoxy substituents, respectively might indicate an activity tolerance to the incorporation of this type of substituents. It was noticed that the activities of compounds **6a**, **6c**, **6d**, **6e** and **6f** bearing 4-methoxyphenoxy moiety were more prominent against hematological cancer rather than other cancer types as shown from data in Figure 11.

In general, the activities of compounds **6g** and **6h** bearing 4-trifluoromethylphenoxy moiety were more prominent against CNS cancer cell lines (Figure 11). Being the only member that bears 2-picolinamido moiety on the 4-posisiton of the pyrimidine core, compound **6h** might engage in an intramolecular hydrogen bonding between the amido NH and the pyridino nitrogen resulting in a pseudo imidazopyridine ring. Owing to this extra rigidification, the geometry of **6h** should be significantly different from other members of the series which might allow it to hit a biomolecule that is exclusively overexpressed in the SF-539 gliosarcoma cell line rationalising its selective lethal activity against this cell line. In addition, this might reflect the involvement of different molecular targets in mediating the activities of these compounds depending on the substitution pattern. Together, these data present compound **6e** and **6h** as potential anticancer lead compounds against diverse cancer types.

#### 3.2.5. *In vitro* assessment of GIT passive permeability assay (PAMPA-GIT assay)

Early assessment of pharmacokinetic parameters of lead compounds is imperative for the development of successful drug discovery programmes. It would help to invest time and effort in developing a worthy series of compounds that possess favourable pharmacokinetic properties and minimise wasting resources on compounds that are unlikely to afford real drugs. Passive permeability across the GIT membrane is an important pharmacokinetic parameter responsible for the absorption of the majority of orally administered drugs. In light of its importance, we checked the permeability of compound **6e** across the GIT membrane employing *in vitro* Parallel artificial membrane permeability assay (PAMPA). The PAMPA assay might be used to simulate passive diffusion across biological barriers such as membranes of GIT and BBB^49^. Simply, the compound of interest is located in a chamber that is separated from another chamber by a suitable membrane, then detecting the amount of the compound reached the other chamber via passive diffusion across the membrane. Compounds showing effective permeability (P_e_) value greater than 1.5 × 10^−6 ^cm/s are considered highly permeable and in case of using GIT-simulating membrane are anticipated to be well-absorbed from GIT. In the course of the conducted assay, 50 μM concentrations of the ultra-highly permeable verapamil and the poorly permeable ranitidine were employed as positive and negative controls, respectively. As shown in Table 4, the measured P_e_ of 12.5 μM concentration of compound **6e** showed an excellent value of 19 × 10^−6 ^cm/s which was 4.4 folds less than the ultra-highly permeable verapamil, but it is 73-folds the P_e_ value of the poorly permeable ranitidine. As the P_e_ of compound **6e** is more than 12-folds the required limit (1.5 × 10^−6 ^cm/s) to confer a good permeability property to a compound, it might be claimed that **6e** is a promising anticancer compound with favourable pharmacokinetic properties considering the anticipated oral absorption.

**Table 4.** *In vitro* measured effective permeability (P_e_) of compound **6e** as well as verapamil and ranitidine using PAMPA-GIT.

Compound	P_e_ (10^−6^ cm/s)±SD
**6e**	19.0 ± 1.1^a^
Verapamil^b^	83.8 ± 2.9
Ranitidine^c^	0.26 ± 0.02

^a^ A 12.5 μM concentration was used and the results are the means of three independent experiments ± standard deviation.

^b^ Verapamil (50 μM) was used as the positive control agent for the PAMPA assay.

^c^ Ranitidine (50 μM) was used as the negative control agent for the PAMPA assay.

#### 3.2.6. Molecular docking simulation study

**Figure 12.**
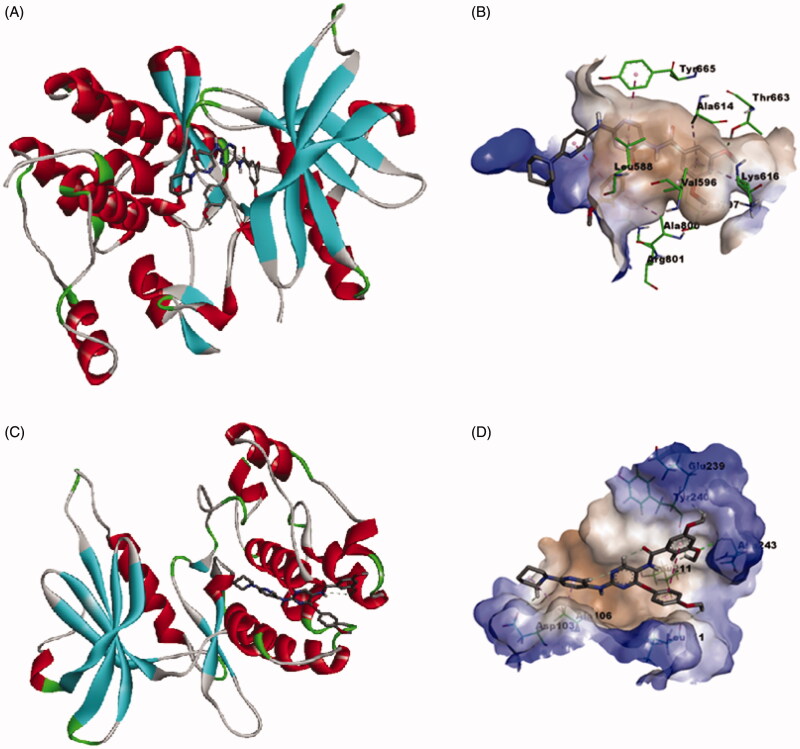
Calculated binding modes and interactions of compound **6e**: (A) Compound **6e** docked within kinase binding site of CSF1R (PDB code: 3KRJ); (B) Predicted interactions of compound **6e** within the binding pocket of CSF1R (PDB code: 3KRJ); (C) Compound **6e** docked within substrate-binding site of DAPK1 (PDB code: 4TXC); (D) Predicted interactions of compound **6e** within the substrate binding pocket of DAPK1 (PDB code: 4TXC).

As noted in the introductory section, the investigated compounds are non-ATP-competitive DAPK1, ATP-competitive CSF1R kinases dual inhibitors. To explore the binding mode of the most active compound **6e** to CSF1R and DAPK1, a molecular docking simulation study was conducted following the previously reported protocol^16^. As Figure 12(A,B) illustrates, the calculated binding mode of compound **6e** into the kinase binding site of CSF1R showed that the pyrimidine ring interacts with the crucial amino acid residue in the hinge region, which is Tyr 665, and the 3,5-dimethoxybenzamide moiety is buried deep into the hydrophobic pocket while the piperidino moiety is protruding out of the pocket towards the solvent region. This calculated binding mode is stabilised with a network of favourable binding interactions involving π–π interactions with Tyr 665, Phe 797, π–alkyl interactions with Leu 588, Val 596, Ala 614, Lys 616, Ala 800, π–cation interaction with Arg 801, and finally hydrogen bonding interaction with Thr 663. As Figure 12(C,D) illustrates for the predicted binding mode within the substrate-binding site of DAPK1, the calculated binding mode showed that compound **6e** fitted within the hydrophobic groove orienting the methoxy substituents on the phenyl groups to face the more hydrophilic region to establish hydrogen bonding interactions with Leu 111, Leu 211, Glu 239, Tyr 240 and Asn 243. In addition to these favourable interactions network, the predicted binding mode establishes π–alkyl and alkyl–alkyl interactions with Ala 106 and hydrogen bonding interaction Asp 103. These results conform with the binding mode of this class of compounds with CSF1R and DAPK1 and provide insights into the binding modes of compound **6e**.

## 4. Conclusion

Heterogeneity and insensitivity of cancers, resistance evolvement, and adverse reactions of anticancer agents create a continuous need to look for new anticancer drugs to meet such unmet clinical needs. In light of this, herein we start from previously identified weak anticancer pyrimidine derivative to design new anticancer agents through hybridisation with 2,4-diarylpyrimidines. The designed series combined the structural features of the starting hit compound and 2-anilino-4-phenoxypyrimidines. The literature reported few compounds belonging to the proposed structures as potential antitauopathies. Provided the additional supportive background, the fact that CSF1R and DAPK1 were found to correlate with cancer diseases promoted these compounds as potential antiproliferative molecules. Synthesis and initial testing of these compounds against leukemic M-NFS-60 mouse cells, which is known to overexpress CSF1R, supported these predictions and suggested the presence of other molecular targets for these compounds in addition to CSF1R and DAPK1. In an attempt to identify molecular targets that cooperate with CSF1R and DAPK1 inhibition to trigger the observed antiproliferative activity, the kinase inhibitory activity of compound **6e** against 14 kinases was assessed. The results showed that **6e** did not disclose the potential inhibition of these kinases. Accordingly, other kinases or non-kinase molecular targets might be involved in mediating the anticancer activity of these compounds. Encouraged by their activity over M-NFS-60 cell line, the synthesised compounds were profiled for their antiproliferative activities against diverse human cancer diseases from nine origins; blood, lung, colon, brain, skin, ovary, renal, prostate and breast. The results showed that compound **6e** (Table 1) was the most promising among the tested compounds showing high and broad-spectrum activity against the tested cancer diseases. Compound **6g** possessing 4-trifluoromethylphenoxy moiety showed broad-spectrum yet lower activity. In addition, a high selective inhibition of some cell lines was found as in the case of compound **6h** which inhibited the growth of SF-539 and SR cell lines by 153.1% and 80.7%, respectively.

The early assessment of important parameters influencing pharmacokinetics is important to avoid wasting precious resources on a lead compound with unfavourable properties, which might cause hurdles in the later development of the clinical candidate(s). Thus, the GIT permeability of compound **6e** was evaluated *in vitro* employing PAMPA assay. The measured good effective permeability (P_e_) of compound **6e** predicted good absorption after oral administration. The conducted molecular docking study of compound **6e** provided insights into the binding modes with CSF1R and DAPK1 and were in conform with the level of CSF1R and DAPK1 inhibitory activity this class of compounds showed. Together, the results present compound **6e** as a promising broad-spectrum antiproliferative lead compound with predicted good GIT permeability that deserves further exploration of its molecular targets and further assessment of its potentiality as a novel polypharmacological anticancer agent.

## Supplementary Material

Supplemental Material
