# Supplementary material for: Reprofiling of pyrimidine-based DAPK1/CSF1R dual inhibitors: identification of 2,5-diamino-4-pyrimidinol derivatives as novel potential anticancer lead compounds
Source: J Enzyme Inhib Med Chem. 2019 Dec 6;35(1):311–24. doi: 10.1080/14756366.2019.1699554 (PMC6913669; doi:10.1080/14756366.2019.1699554)
Supplement: Supplemental Material [file IENZ_A_1699554_SM0421.pdf]

Reprofiling of pyrimidine-based DAPK1/CSF1R dual inhibitors: Identification of 2,5-diamino-4-pyrimidinol derivatives as novel potential anticancer lead compounds

Ahmed Karam Farag[a,*], Ahmed H.E. Hassan[b], Buyng Sun Ahn[c,d], Ki Duk Park[d,e], Eun Joo Roh[c,d,*]

[a] *RI Translational Research Team, Division of Applied RI, Korea Institute of Radiological and Medical Sciences (KIRAMS), Seoul, 01812, Republic of Korea.*

[b] *Department of Medicinal Chemistry, Faculty of Pharmacy, Mansoura University, Mansoura, 35516, Egypt.*

[c] *Chemical Kinomics Research Center, Korea Institute of Science and Technology (KIST), Seoul 02792, Republic of Korea.*

[d] *Division of Bio-Medical Science &Technology, KIST School, University of Science and Technology, Seoul, 02792, Republic of Korea.*

[e] *Convergence Research Center for Diagnosis, Treatment and Care System of Dementia, Korea Institute of Science and Technology (KIST), Republic of Korea.*

[*]Corresponding authors:

Ahmed Karam Farag (email address: ahmed@kirams.re.kr; postal address: RI Translational Research Team, Division of Applied RI, Korea Institute of Radiological and Medical Sciences, 75 Nowon-ro, Nowon-gu, Seoul, 01812, Republic of Korea).

Eun Joo Roh (email address: r8636@kist.re.kr; postal address: Korea Institute of Science and Technology, Materials and Life Science Research Division, Chemical Kinomics Research Center, Hwarang-ro 14-gil 5, Seongbuk-gu, Seoul, 02792, Republic of Korea).

Contents

1. **Representative spectral data for the novel compounds.**

2. **Biological experimental data**

    2.1. *In vitro* antiproliferative assay using M-NFS-60 cell lines

    2.2. PAMPA-GIT assay

1. Representative spectral data for the novel compounds.

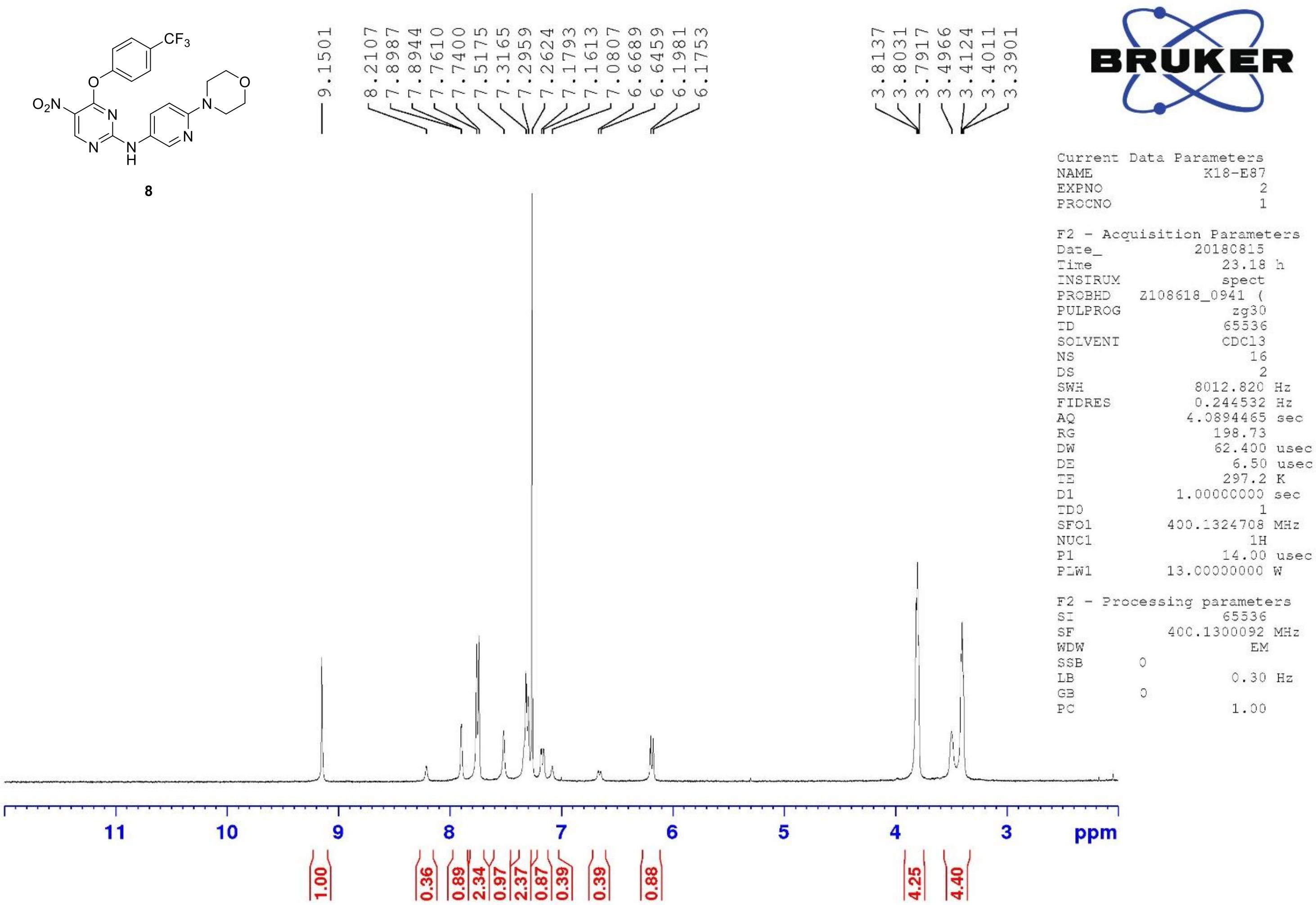

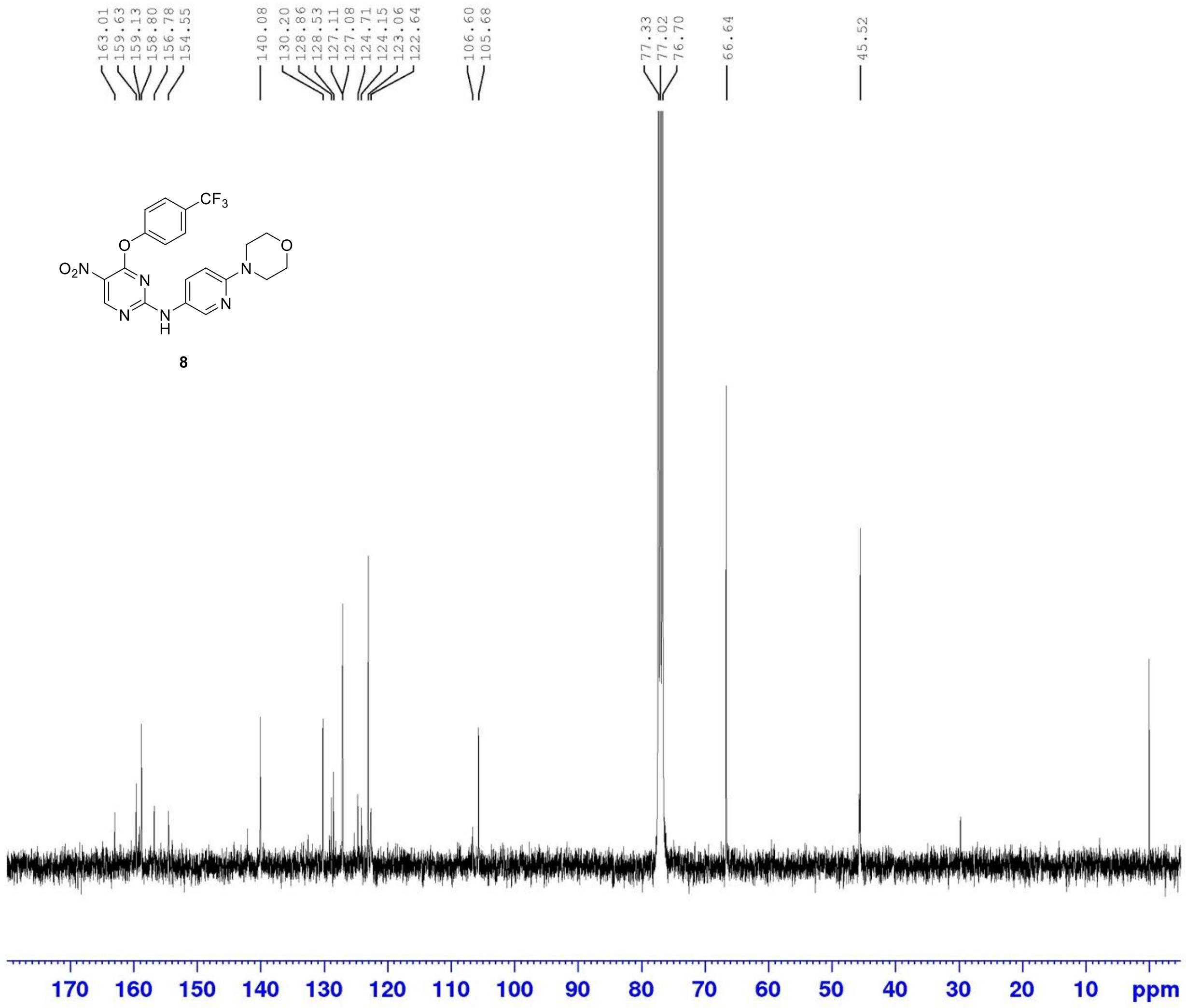

8

Current Data Parameters
NAME E87_C13_16_10
EXPNO 1
PROCNO 1

F2 - Acquisition Parameters
Date_ 20191016
Time 8.46
INSTRUM spect
PROBHD 5 mm PABBO BB/
PULPROG zgpg30
TD 65536
SOLVENT CDCl3
NS 10900
DS 4
SWH 24038.461 Hz
FIDRES 0.366798 Hz
AQ 1.3631488 sec
RG 190.62
DW 20.800 usec
DE 6.50 usec
TE 299.9 K
D1 2.00000000 sec
D11 0.03000000 sec
TD0 1

======== CHANNEL f1 ========
SFO1 100.6429474 MHz
NUC1 13C
P1 10.00 usec
PLW1 52.00000000 W

======== CHANNEL f2 ========
SFO2 400.2116008 MHz
NUC2 1H
CPDPRG[2 waltz16
PCPD2 90.00 usec
PLW2 12.00000000 W
PLW12 0.35777000 W
PLW13 0.28979000 W

F2 - Processing parameters
SI 32768
SF 100.6328850 MHz
WDW EM
SSB 0
LB 1.00 Hz
GB 0
PC 1.40

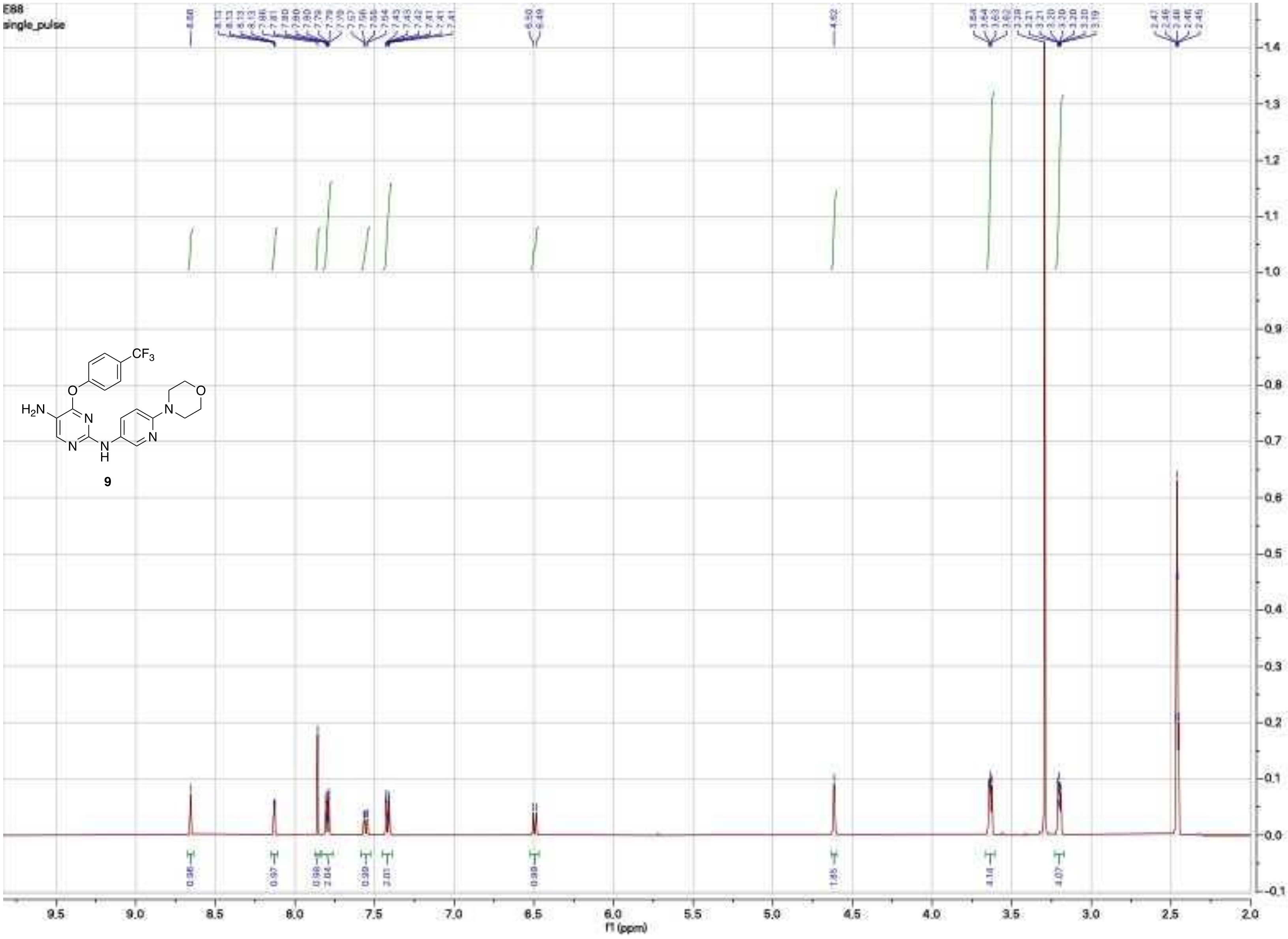

E88
single_pulse
H2N
CF3
O
N
NH
9
f1 (ppm)

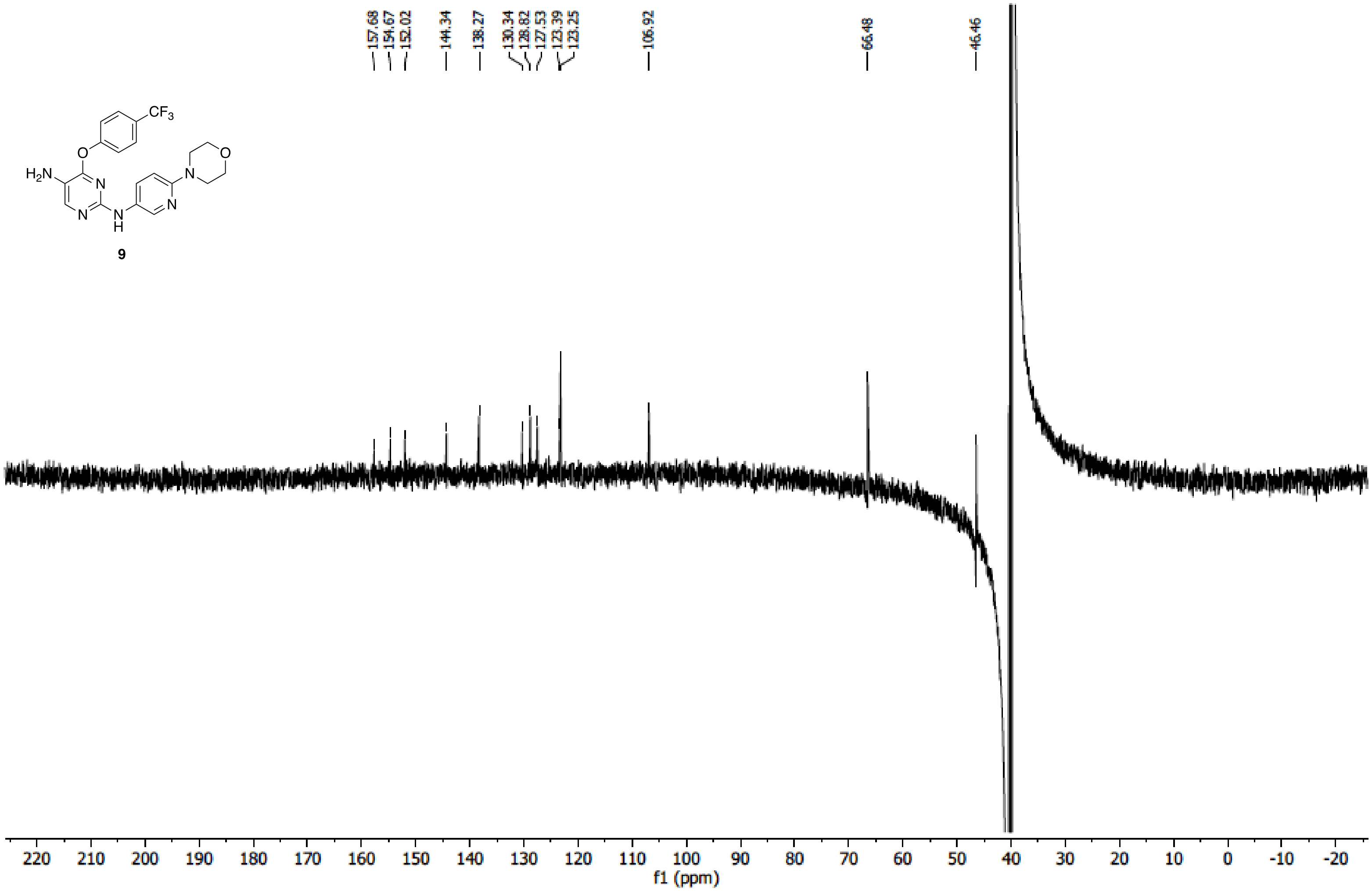
CF3
O
H2N
N
N
N
H
N
N
O
157.68
154.67
152.02
144.34
138.27
130.34
128.82
127.53
123.39
123.25
106.92
66.48
46.46
220 210 200 190 180 170 160 150 140 130 120 110 100 90 80 70 60 50 40 30 20 10 0 -10 -20
f1 (ppm)

9

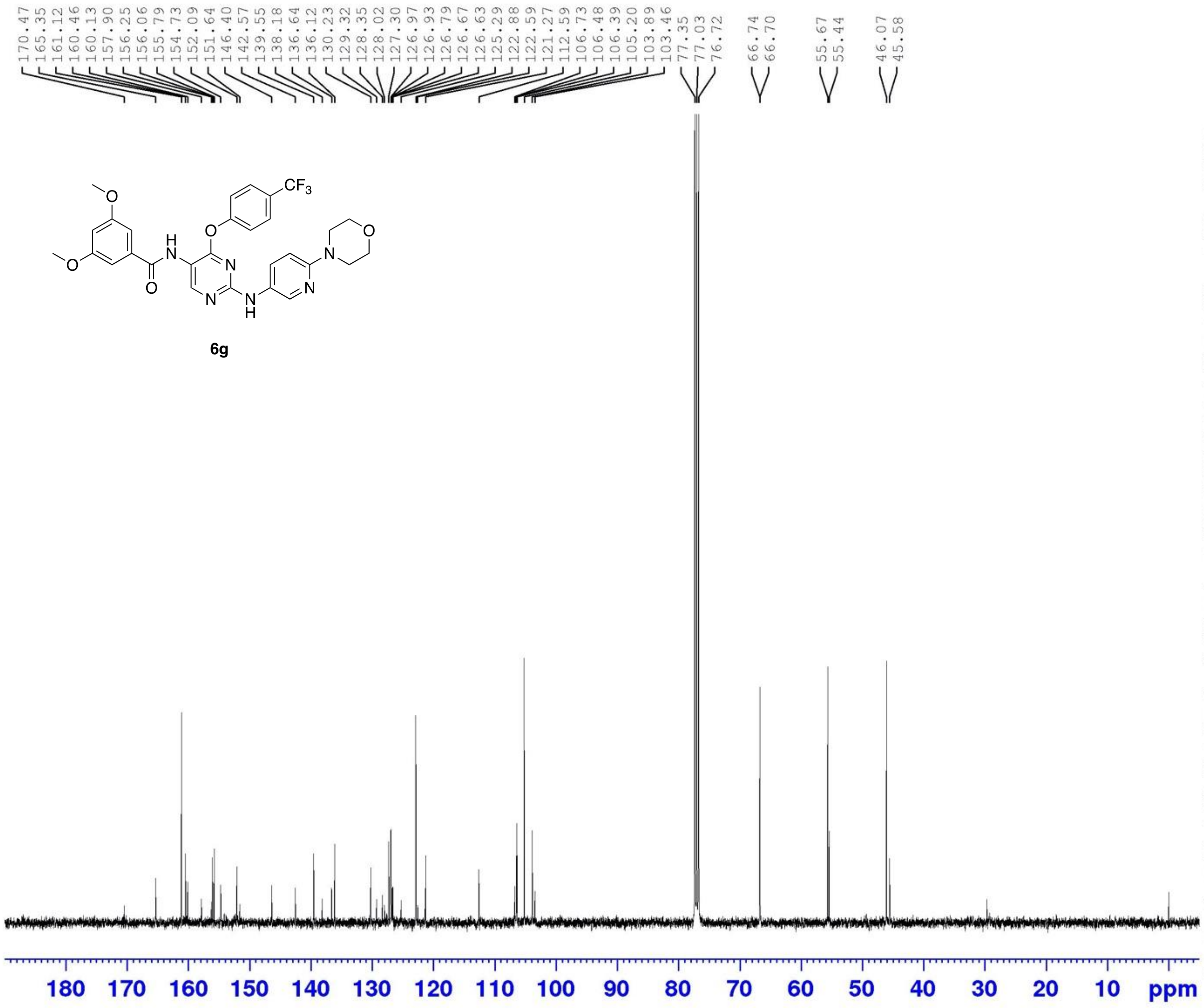

6g

BRUKER

```
Current Data Parameters
NAME          K18-E89-C13
EXPNO                   1
PROCNO                  1

F2 - Acquisition Parameters
Date_            20180810
Time                 1.12
INSTRUM             spect
PROBHD   5 mm PABBO BB/
PULPROG           zgpg30
TD                  65536
SOLVENT             CDCl3
NS                   1024
DS                      4
SWH             24038.461 Hz
FIDRES           0.366798 Hz
AQ              1.3631488 sec
RG                 190.62
DW                 20.800 usec
DE                   6.50 usec
TE                  298.2 K
D1             2.00000000 sec
D11            0.03000000 sec
TD0                     1

======== CHANNEL f1 ========
SFO1          100.6429474 MHz
NUC1                  13C
P1                  10.00 usec
PLW1          52.00000000 W

======== CHANNEL f2 ========
SFO2          400.2116008 MHz
NUC2                   1H
CPDPRG[2          waltz16
PCPD2               90.00 usec
PLW2          12.00000000 W
PLW12          0.35777000 W
PLW13          0.28979000 W

F2 - Processing parameters
SI                  32768
SF            100.6328860 MHz
WDW                    EM
SSB      0
LB                   1.00 Hz
GB       0
PC                   1.40
```

6g

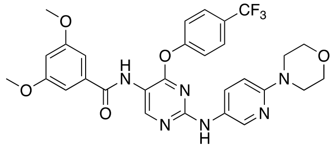

6g

Molecular formula: $C_{29}H_{27}F_3N_6O_5$
Exact mass: 596.1995
150.0577 (4+)
199.7410 (3+)
299.1076 (2+)
597.2073 (1+)

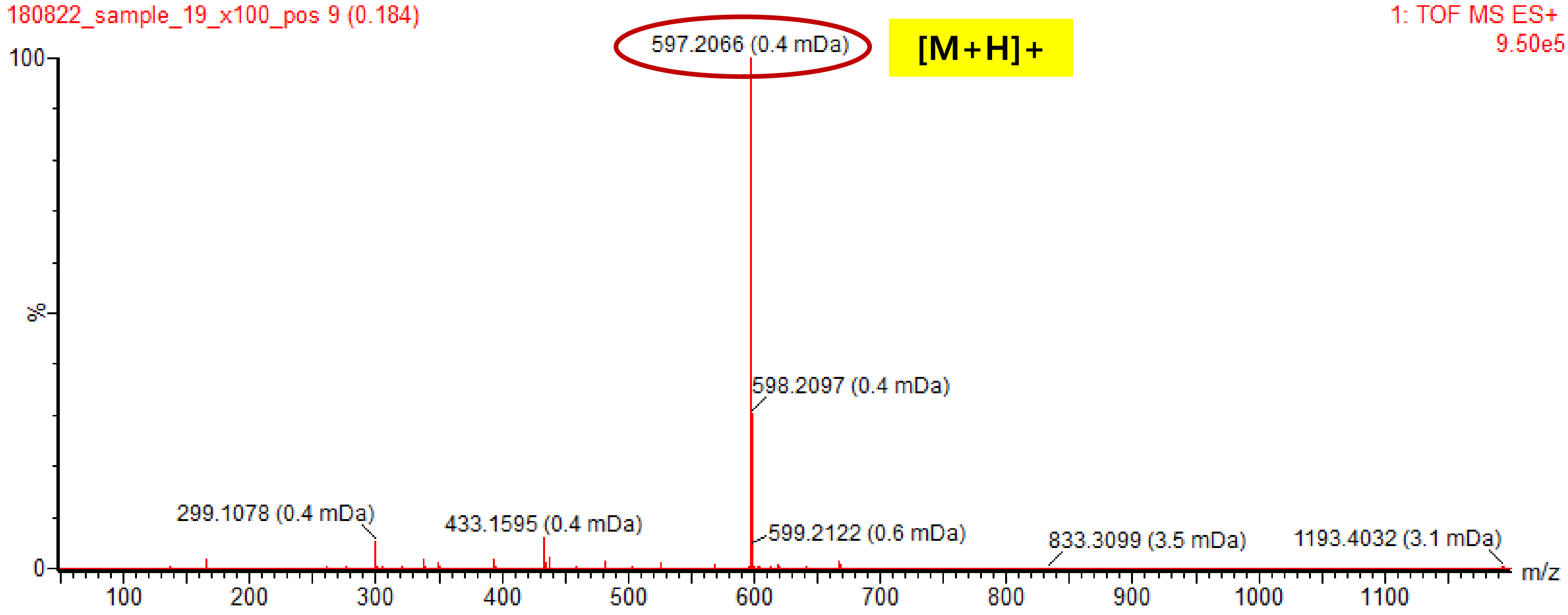

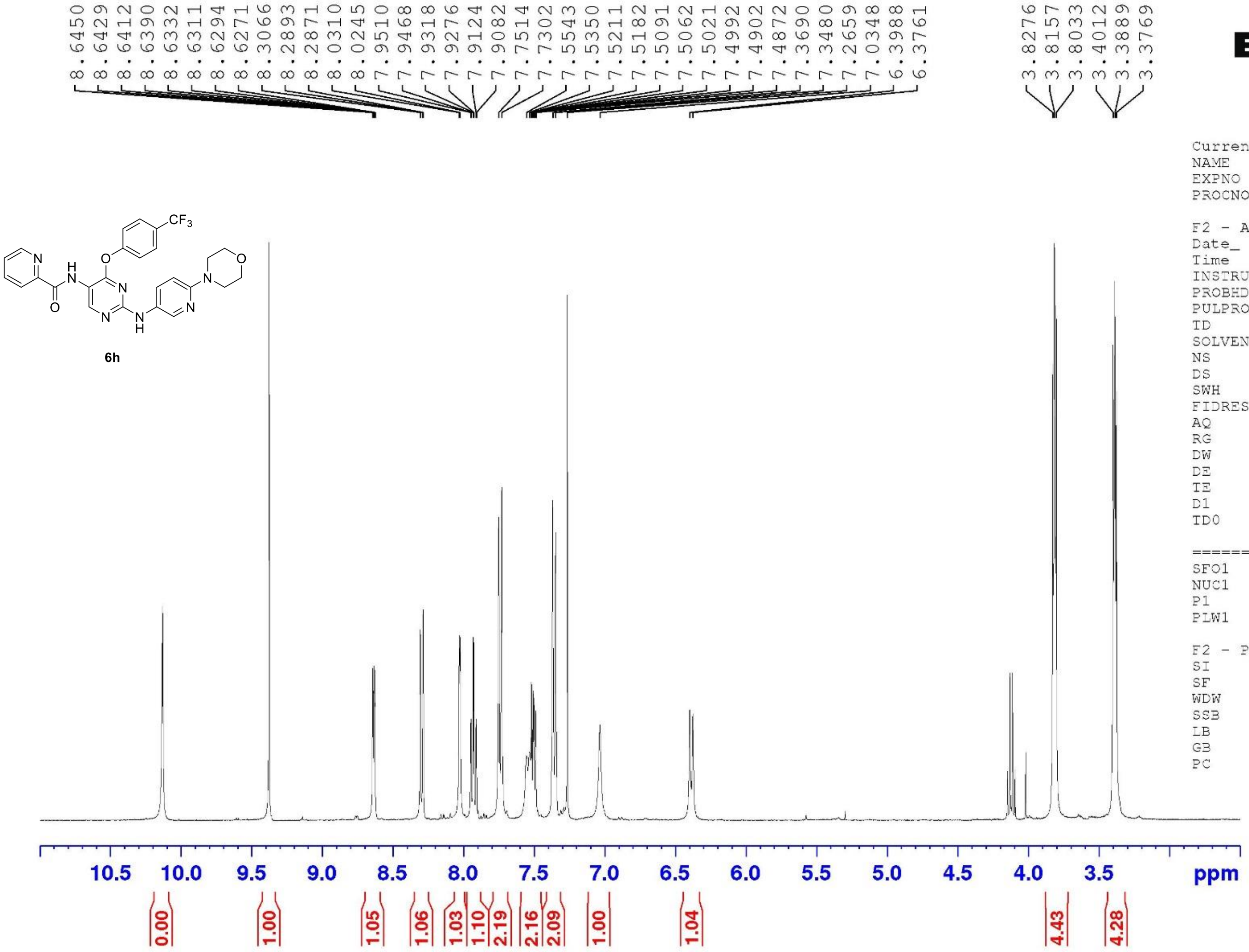

6h

```
Current Data Parameters
NAME                 K18-E90
EXPNO                      1
PROCNO                     1

F2 - Acquisition Parameters
Date_               20180810
Time                    1.33
INSTRUM                spect
PROBHD   5 mm PABBO BB/
PULPROG                 zg30
TD                     65536
SOLVENT                CDCl3
NS                        16
DS                         2
SWH                 8012.820 Hz
FIDRES              0.122266 Hz
AQ                 4.0894465 sec
RG                    190.62
DW                    62.400 usec
DE                      6.50 usec
TE                     297.1 K
D1                1.00000000 sec
TD0                        1

======== CHANNEL f1 ========
SFO1             400.2124715 MHz
NUC1                      1H
P1                     15.54 usec
PLW1             12.00000000 W

F2 - Processing parameters
SI                     65536
SF               400.2100110 MHz
WDW                       EM
SSB      0
LB                      0.30 Hz
GB       0
PC                      1.00
```

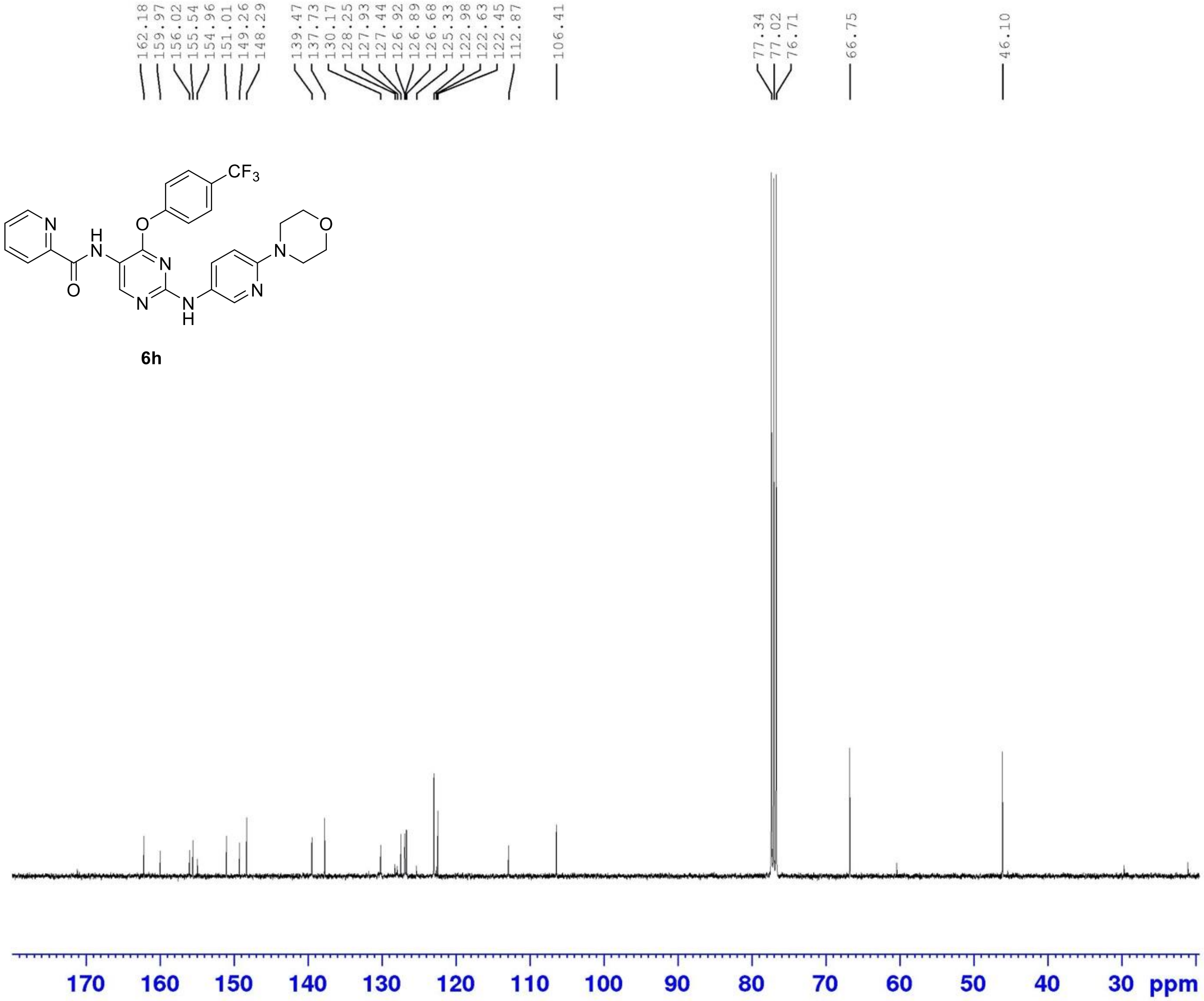


```
Current Data Parameters
NAME          K18-E90-C13
EXPNO                   1
PROCNO                  1

F2 - Acquisition Parameters
Date_            20180810
Time                 2.36
INSTRUM             spect
PROBHD   5 mm PABBO BB/
PULPROG            zgpg30
TD                  65536
SOLVENT             CDCl3
NS                   1024
DS                      4
SWH             24038.461 Hz
FIDRES           0.366798 Hz
AQ              1.3631488 sec
RG                 190.62
DW                 20.800 usec
DE                   6.50 usec
TE                  298.3 K
D1             2.00000000 sec
D11            0.03000000 sec
TD0                     1

======== CHANNEL f1 ========
SFO1         100.6429474 MHz
NUC1                  13C
P1                  10.00 usec
PLW1          52.00000000 W

======== CHANNEL f2 ========
SFO2         400.2116008 MHz
NUC2                   1H
CPDPRG[2          waltz16
PCPD2               90.00 usec
PLW2          12.00000000 W
PLW12          0.35777000 W
PLW13          0.28979000 W

F2 - Processing parameters
SI                  32768
SF            100.6328860 MHz
WDW                    EM
SSB      0
LB                   1.00 Hz
GB       0
PC                   1.40
```

6h

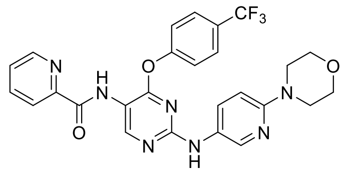

6h

Molecular formula: $C_{26}H_{22}F_3N_7O_3$
Exact mass: 537.1736
135.3012 (4+)
180.0657 (3+)
269.5946 (2+)
538.1814 (1+)

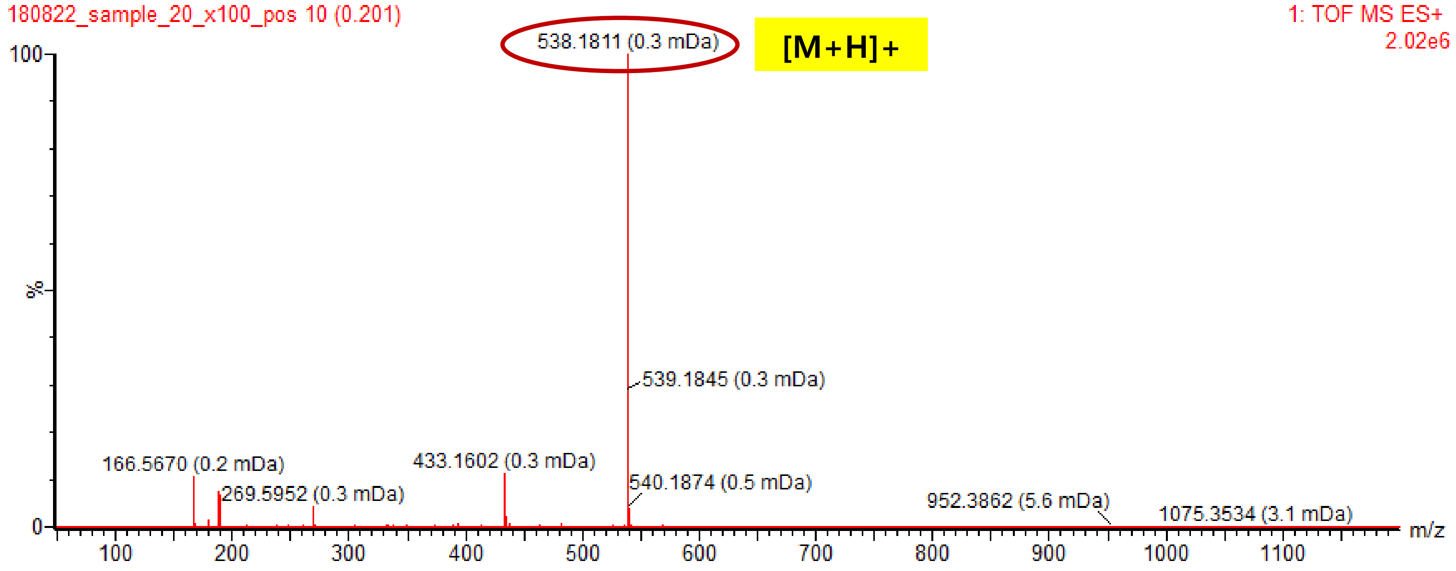

2. **Biological experimental data**

2.1.In vitro antiproliferative assay using M-NFS-60 cell lines

Materials:

Test compounds and staurosporine were dissolved in DMSO in 10 mM stock. Staurosporine was purchased from Sigma-Aldrich (Saint Louis, MI). Cell Titer-Glo® 2.0 Luminescent cell viability assay reagent was purchased from Promega (Madison, WI). M-NFS-60 cell line was purchased from American Type Culture Collection (Manassas, VA). Cell culture media were as follow: RPMI-1640 + 10% FBS + 20 ng/ml M-CSF. All media are supplemented with 100 µg/mL of penicillin, and 100 µg/mL of streptomycin. Cultures were maintained at 37°C in a humidified atmosphere of 5% CO_2 and 95% air.

Procedures:

1. Test compounds and staurosporine were diluted in DMSO solution with 10-dose and 3-fold dilutions in a source plate starting at 10 mM.
2. 25 nL of each test compound or staurosporine was delivered from the source plate to each well of the 384-well cell culture plates by Echo 550.
3. 25 µL of culture media containing 2000 M-NFS-60 cells was added to each of the wells in duplicates of the cell culture plate.
4. The cells were incubated with the compounds at 37°C, 5% CO_2 for 72 hours.

5. 25 µL of Cell Titer Glo 2.0 reagent was added to each well.
6. The contents were mixed on an orbital shaker for 2 min and incubated at room temperature for 15 min to stabilize luminescent signal.
7. Luminescence was recorded by Envision 2104 Multilabel Reader (PerkinElmer, Santa Clara, CA). The number of viable cells in culture was determined based on quantitation of the ATP present in each culture well.
8. The IC_{50} curves were plotted and IC_{50} values were calculated using the GraphPad Prism 4 program based on a sigmoidal dose-response equation.

*2.2.*PAMPA-GIT Assay [1-3]

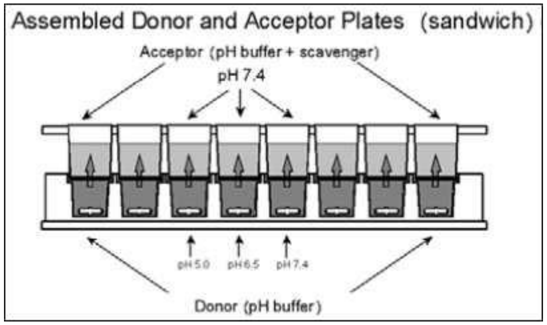

The effective permeabilities of compound **6e**, and two reference drugs (Verapamil and Ranitidine) were assessed using Double-Sink™ (Pion, GI-PAMPA) via applying a donor 96 well-plate microtitre and an acceptor 96-well filter plate to form a sandwich as illustrated in the figure. Thus, each well is divided into two chambers, separated by a GI-PAMPA membrane. 10 mM DMSO stock

solutions of the tested compounds and reference drugs were prepared and diluted with Prisma buffer solution (pH = 7.4) to provide solutions of 12.5 (**6e**) and 50 μM (reference drugs) concentrations, which were placed in the wells of the donor plate. The acceptor plate containing buffer was placed and allowed to incubate for 4 h at 26 °C. The acceptor plate was removed and the concentration of compounds in the acceptor, the donor, and the reference wells were measured by UV (230–498 nm). The effective permeability (P_e) of each compound was calculated using Pion PAMPA Explorer software (version 3.8) applying calculation basis double sink (acceptor+donor+membrane).The following P_e ranges were established: low penetration of GIT ($P_e < 1.5 \times 10^{-6}$), high penetration of GIT ($P_e > 1.5 \times 10^{-6}$).

References:

[1] M. Kansy, F. Senner, K. Gubernator, Physicochemical High Throughput Screening: Parallel Artificial Membrane Permeation Assay in the Description of Passive Absorption Processes, Journal of Medicinal Chemistry, 41 (1998) 1007-1010.

[2] High-Throughput Measurements of Solubility Profiles, in: Pharmacokinetic Optimization in Drug Research.

[3] A. Avdeef, M. Strafford, E. Block, M.P. Balogh, W. Chambliss, I. Khan, Drug absorption in vitro model: filter-immobilized artificial membranes: 2. Studies of the permeability properties of lactones in Piper methysticum Forst, Eur. J. Pharm. Sci., 14 (2001) 271-280.
